# A systematic scoping review for decolonial public and global health: Indigenous frameworks and models of wellbeing from Turtle Island and Moananuiākea

**DOI:** 10.3389/fpubh.2026.1809539

**Published:** 2026-07-16

**Authors:** Joanne Qina‘au, Aubrey Yanger Mariano, Melissa K. Kahili-Heede, Selah Qasataisqaq Kone, Caleb Rivera, Cerila Rapadas, Shayla Chatto, Valerie J. Clack, Finley Ngarangi Johnson, Michelle Johnson-Jennings, Mapuana C. K. Antonio

**Affiliations:** 1 Osher Center for Integrative Health, University of California, San Francisco, San Francisco, CA, United States; 2 Papa Ola Lōkahi, Honolulu, HI, United States; 3 Office of Public Health Studies, University of Hawai‘i at Mānoa, Honolulu, HI, United States; 4 Human Development & Community Health, Montana State University, Bozeman, MT, United States; 5 Department of Psychology, University of Hawai‘i at Mānoa, Honolulu, HI, United States; 6 Early Childhood Multicultural Education, Navajo Technical University, Crownpoint, NM, United States; 7 Pilina Center for Wellbeing, Honolulu, HI, United States; 8 Te Herenga Waka - Victoria University of Wellington, Te Whanganui-a-Tara (Wellington), Aotearoa, New Zealand; 9 Indigenous Wellness Research Institute/IWRI, School of Public Health, School of Social Work, University of Washington, Seattle, WA, United States; 10 Native Hawaiian and Indigenous Health, Office of Public Health Studies, University of Hawai‘i at Mānoa, Honolulu, HI, United States

**Keywords:** decolonial, framework, Indigenous, model, native, Pacific, Turtle Island, wellbeing

## Abstract

**Background:**

Equitable approaches to public and global health require grounding in diverse ontologies of wellbeing and decolonial theory. Indigenous wellbeing models and frameworks embed holistic values that support ecological thriving. In contrast, Western approaches often individualize and compartmentalize wellbeing, narrowing public and global health practice and contributing to persistent disparities. Despite the proliferation of Indigenous-developed frameworks, no scoping review has synthesized contributions in this area. This review aimed to identify models, frameworks, and theories of wellbeing developed by Indigenous communities and scholars from Turtle Island (North America) and Moananuiākea (the Pacific) to inform more culturally safe and decolonial approaches to public and global health.

**Methods:**

Guided by the PRISMA-ScR framework, both peer-reviewed publications and grey literature were sourced from five databases (PubMed, Web of Science, EBSCO Academic Search Complete, CINAHL, and ProQuest). Additional articles were hand-sourced. Included articles posed an original model, framework, or theory of wellbeing, developed for Indigenous people, families, or communities, by Indigenous authors from those respective lands in Turtle Island or Moananuiākea.

**Results:**

A total of 1,473 database references and 89 additional references were identified for screening. After 61 full-text reviews, 33 manuscripts were included for analysis. Twenty-one publications originated from Moananuiākea (64%) and 12 originated from Turtle Island (36%). The most common method used to develop the models and frameworks was qualitative (52%). Synthesis of the data yielded seven themes: (1) holistic integration of all life domains; (2) spirituality and the ongoing presence of ancestors; (3) culture and preservation of tradition; (4) collective wellbeing and relationality; (5) reciprocal relationship with land as a source of identity, health, and responsibility; (6) use of place-specific Indigenous metaphors and language; and (7) empowerment and self-determination.

**Conclusion:**

Indigenous communities globally are calling for a shift from conceptual discussion of wellbeing toward tangible application in public and global health systems. These findings hold several implications for decolonial theory-development and more equitable health systems. Future research should build on work which moves Indigenous wellbeing frameworks from theory to action.

## Introduction

Indigenous approaches to health and wellbeing are deeply relational, holistic, spiritual, and place based. Across Turtle Island (North America) and Moananuiākea (the Pacific), Indigenous peoples have maintained balanced ecological wellbeing through reciprocal pilina (relationship, connection) with land, ancestors, and the spiritual domain for millennia (1, 2). In contrast, dominant Eurocentric conceptions of wellbeing typically compartmentalize elements, often emphasizing individualistic and hedonistic constructs (3, 4). While Eurocentric approaches offer important insights, they rarely capture the interconnected spiritual, ecological, and collective aspects of wellbeing emphasized in Indigenous worldviews (5). Historically, a focus on individualistic and hedonistic wellbeing has contributed to settler-colonial stress (6), further exacerbating disparities for Indigenous peoples in both physical and mental health domains (7–10).

Public health systems have increasingly recognized wellbeing as central to health promotion, policy, and service delivery. For example, the United States *Healthy People* initiative includes wellbeing measures such as life satisfaction and quality of life (11). Yet, such indicators risk narrowing wellbeing to individual-level outcomes, disconnected from history, culture, and environment (12). Indigenous frameworks position wellbeing within collective, spiritual, and ecological contexts, recognizing resilience and survival in the face of colonialism (13–15). These frameworks offer insights to Indigenous health while reshaping public health toward greater cultural safety (16) and sustainability.

Exemplars demonstrate the practical effectiveness of models and frameworks grounded in Indigenous ways of knowing. In Alaska, the tribally owned Southcentral Foundation Nuka System of Care reorganizes healthcare around Indigenous values and patient ownership, producing significant improvements in access, preventive care, and outcomes (17, 18). Similar efforts are underway in Canada (19) and Aotearoa (20) where Indigenous worldviews of health are foundational or integrated in clinical settings. These cases demonstrate that when health systems are intentionally designed around Indigenous ways of being, they enable relational, holistic, and culturally safe approaches to care that produce more effective, equitable, and sustainable wellbeing outcomes for all.

Across many Indigenous worldviews, the health of people is inseparable from the health of land and ecosystems, positioning ecological wellbeing as foundational rather than ancillary to human wellbeing. Indigenous frameworks such as the Samoan Fonofale (21), Tokelauan Te Vaka Atafaga (22), the United Houma Nation’s Uma Hochokma Framework (23), and the Indigenous Traditional Ecological Framework and Indigenous eco-relational engagement (24) operationalize this orientation by foregrounding reciprocity and interdependence between human and environmental health. This orientation contrasts Eurocentric paradigms, which often compartmentalize human and environmental health, framing their connection through linear cause-and-effect models rather than reciprocal interdependence (25). When land and ecological health are not centered, issues such as the climate crisis exacerbate poor physical, emotional, economic, social, and spiritual health (26, 27), particularly for Indigenous communities (28, 29). Thus, Indigenous ecological models and frameworks of wellbeing are both timely and necessary in reorienting the field toward ecological and relational views to advance health equity (30).

Finally, Indigenous models of wellbeing provide guidance to inform discourse and action for decolonial efforts in global and public health. Decolonial theory and practice do not often acknowledge Indigenous ontology, epistemology, axiology, and praxis and instead aim for a more general restructuring of power imbalances to center “local” decision-making (31). Indigenous health is marginalized and scholarship addressing colonialism is surprisingly absent of Indigenous representation (31, 32). This demonstrates the co-optation of decolonial efforts and further contributes to the erasure of Indigenous lifeways. Reviews of decolonialism call for increased shared decision-making with Indigenous communities, long-term funding mechanisms for Indigenous-led research and health efforts, and more Indigenous authorship and leadership (33, 34). However, inclusion without transfer of power is a continuation of coloniality, which inevitably upholds inequitable health outcomes. Global and public health cannot be decolonized without Indigenous self-determination and representation from theory to practice. Indigenous models of wellbeing provide guidance for how that might be achieved.

### Rationale

The present scoping review contributes to an understanding of Indigenous wellbeing by exploring relevant models and frameworks from across Turtle Island and Moananuiākea. These regions were chosen given the authors’ positioning (see Supplementary material A): based in and with ancestral ties across Turtle Island and Moananuiākea, representing a bridge between these regions physically, politically, and culturally. A review focused on both regions allows for a more robust understanding of Indigenous wellbeing models and frameworks that illuminate common patterns and important distinctions shaped by specific cultural, historical, and environmental contexts. While models and frameworks of Indigenous wellbeing no doubt exist in other regions, this review is limited to two regions to ensure feasibility and thoroughness of analysis. Despite the growing number of Indigenous-developed wellbeing models, there has been no comprehensive review synthesizing their features across these two regions. This gap perpetuates the privileging of Eurocentric theories in public and global health and limits understanding of Indigenous contributions to theory and practice. Addressing this gap brings equity to the forefront and acknowledges that Indigenous ontologies, epistemologies, and axiologies must be centered as valid sources of knowledge (2, 35), informing practice in ways that bring greater equity and sustainability for all.

### Objectives

Guided by Indigenous methodologies, this scoping review has one primary aim and one secondary aim:

Primary aim: To map Indigenous models and frameworks of wellbeing from Turtle Island and Moananuiākea, describing their genealogies and constructs.

Secondary aim: To contribute to decolonial theory-building in public health by centering Indigenous ontologies, epistemologies, axiologies, and praxes as valid and vital.

By analyzing these frameworks and synthesizing aspects of their uses in public health, we seek to contribute to a growing movement to decolonize public health, celebrate Indigenous excellence, and recognize Indigenous models of wellbeing as foundational for health equity and global sustainability (30, 36). While we recognize the distinctions between models and frameworks, we use the terms interchangeably throughout this manuscript.

## Methods

### Research approach

Given the history of unethical research practices and mistrust in academic institutions (37), and to capture the deeply nuanced aspects of the topic it is important to approach research on Indigenous wellbeing using Indigenous research methods. Our review process integrated intentional strategies to ensure the inclusion of Indigenous concepts, languages, and relational understandings that are often excluded or misunderstood in conventional research. We used the transformative paradigm, an Indigenous approach to research, which explicitly names the transformation of systems to support more equitable power dynamics as a primary goal. Research guided by this paradigm contributes to social transformation with a unique approach to all four parts of a theoretical paradigm: ontology, epistemology, axiology, and methodology (38). This approach provides a critical scientific lens which overcomes limitations in positivist and constructivist paradigms that are rooted in colonial epistemologies (39).

This review uses an Indigenous Approach, conducted by and for Indigenous communities (37, 40). Given this approach, all members of the research team are Indigenous, from Turtle Island or Moananuiākea (see Supplementary material A for positionality statements). This review is positioned as part of an effort to decolonize public and global health (41), situated as Indigenous resistance, centering Indigenous voices and worldviews (42). Its orientation to decolonizing explores generating knowledge with and from within, aiming for epistemic justice (43, 44) by grounding subjectivities of knowledge in land and bodies, centers of emancipation (45).

We approached this research as an act of ceremony, using meeting times to nourish relationships through *talk story*, exploring reciprocal and community benefits of our efforts, and, ultimately, support our individual and collective journeys of enlightenment (2). The relationships built during this process are honored as being equally important as the outcome of this review. By beginning meetings with protocol and meditations from our respective cultures, we honored the inherent spirituality in life itself, which includes our research huakaʻi (journey). This approach fosters a future where Indigenous methodologies are not only recognized and respected but integral to ethical, effective, and globally relevant public health endeavors, contributing to justice and sovereignty in knowledge development for generations to come.

### Protocol and registration

The scoping review team included subject-matter specialists in the disciplines of Native Hawaiian and Indigenous health, wellbeing, clinical and community psychology, public health, culturally sustaining education, and library and information sciences. The conceptual framing and research questions of this paper stemmed from ongoing work and shared research by the first and last author. The inclusion and exclusion criteria were defined collectively by the research team through ongoing discussions and preliminary review of the literature. The scoping review was conducted in accordance with the JBI methodology for scoping reviews (46). In addition, the methods for this scoping review are reported in accordance with the PRISMA guidelines extension for scoping reviews (47). The research team also registered a protocol for the review in Open Science Framework.^1^ The protocol was registered in September 2023 and detailed information around the review, populations (e.g., Indigenous communities from Turtle Island and Moananuiākea), variables, and primary focus (e.g., models and frameworks of wellbeing).

### Eligibility criteria

Inclusion criteria were articles that presented on (1) models, frameworks, and theories; (2) wellbeing/wellness/quality of life; and (3) Indigenous adults, families, or communities in Turtle Island and Moananuiākea. Exclusion criteria were articles that focused solely on health (exclusive of wellbeing), on model homographs (e.g., role model), wellbeing models specific to COVID-19 and other diagnoses, environmental wellbeing, specific outcomes (e.g., cancer); and populations that were non-Indigenous, youth, or from geographies outside Turtle Island and Moananuiākea. The decision to exclude articles exclusively focused on “health” was made to maintain a focused and feasible scope, as their inclusion would have substantially expanded the volume of literature. Nevertheless, several authors used the terms “health” and “wellbeing” interchangeably.

### Search strategy

The search strategy aimed to locate published and unpublished studies related to wellbeing frameworks for Indigenous people of Turtle Island and Moananuiākea. JQ and MK-H ran preliminary searches based on known frameworks to identify additional articles on the topic. A full search strategy was then developed based on words contained in the titles and abstracts of relevant articles as well as the index terms used to describe the articles (see Supplementary material B). The search strategy was adapted for each database and/or information source. The reference list of all included sources was screened for additional studies.

**Figure 1:**
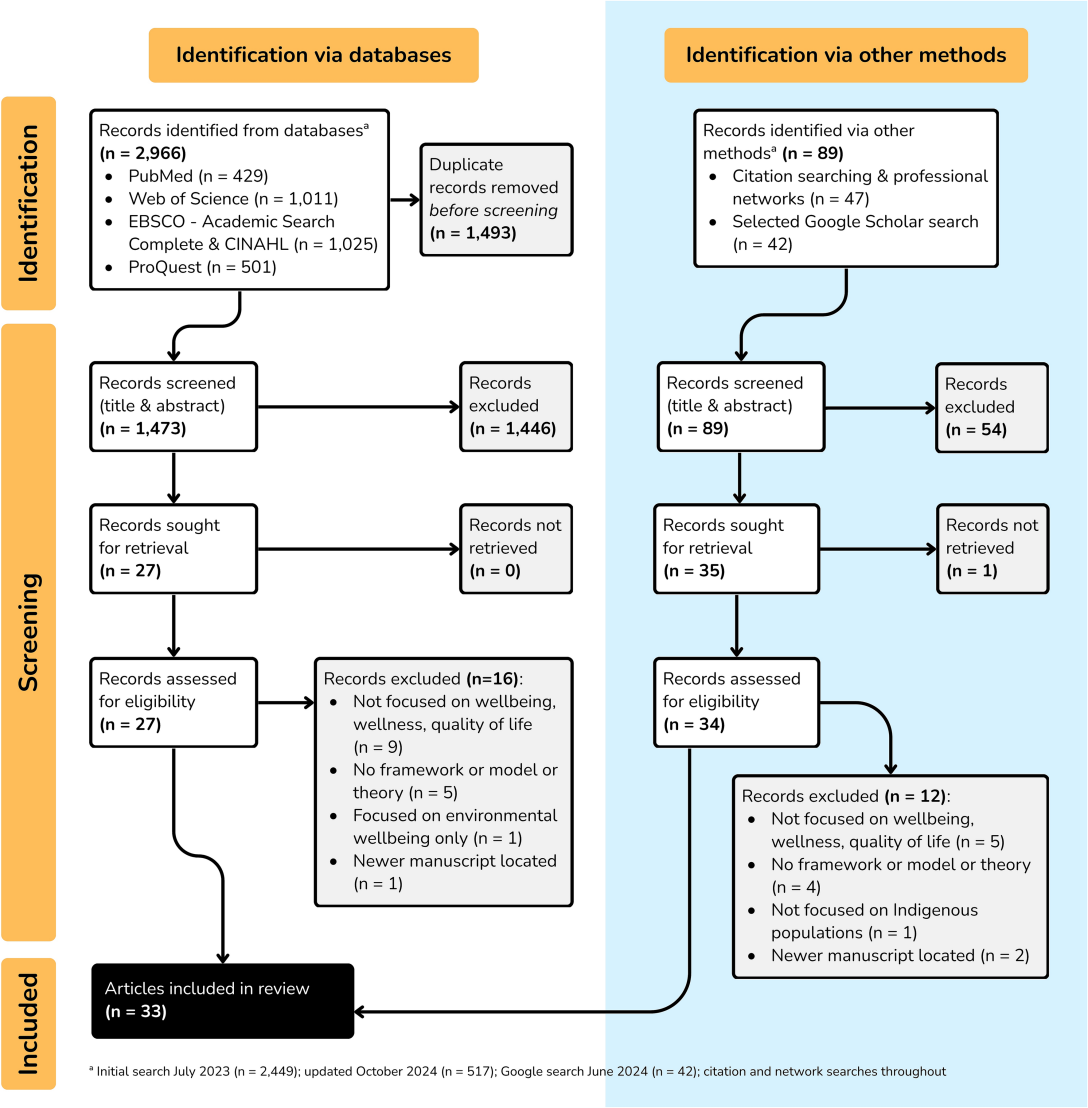
PRISMA flow diagram.

The initial search for this review was conducted in July 2023 using the following: Academic Search Complete and Cumulative Index to Nursing and Allied Health Literature (CINAHL) via EBSCOhost, ProQuest databases, PubMed/Medline, and Web of Science Core Collection. We searched health-related and multidisciplinary databases due to the interdisciplinary nature of this topic. We used a combination of wellbeing, framework, and Indigenous peoples’ terms (refer to Supplementary material B). Search terms for framework and Indigenous peoples were combined using proximity operators. These results were limited to English language and peer-reviewed articles, without date restrictions. The initial database search yielded 2,449 articles, and this search was re-run in October of 2024 resulting in 517 additional articles (see Figure 1, PRISMA Flow Diagram).

Lastly, to ensure a comprehensive approach, authors emailed listservs and specific research teams, resulting in 47 additional articles. One Indigenous scholar from the research team’s networks recommended using Google Scholar to capture grey literature. A preliminary search was conducted to assess the number of new and relevant articles retrieved by this database. The search yielded over 530 k articles and 42 were included for screening in this study (see Supplementary material B). Due to an excess of search results, the research team determined this database would require a separate, independent study. Altogether, 1,562 unique records were identified across all sources after duplicate removal. Of these, 1,473 records from database searching were screened at the title and abstract stage; the 89 records identified via other methods were advanced directly to full-text review.

#### Selection process (first-line screening)

Using a collaborative team approach, Rayyan was used to manage articles captured in the initial database search, to apply first-line screening based on the inclusion/exclusion criteria, and to ensure quality assurance. Two reviewers independently screened eligible studies for selection. The research team used a Decision Tree (see Supplementary material C) to determine inclusion/exclusion criteria not previously defined and to resolve “maybes” from the initial round of abstract reviews. The “maybes” consisted of abstracts where discrepancies in decision existed between the two reviewers assigned to the abstract; a third reviewer or the research team at large discussed “maybes” until we reached consensus.

In total, 2,966 records were obtained from all databases. An additional 47 were included from citation searching and professional networks and 42 were identified viaGoogle Scholar. After duplicate removal, 1,473 unique records were screened by two independent reviewers. There were 56 conflicts (3.8%), resulting in a percent agreement of 96.2%. Disagreements were resolved by consensus. Upon completion of the initial screening phase (title and abstract review), 27 articles from databases and 34 from professional networks and Google Scholar were advanced to the Full Text Review stage.

Articles included for Full Text Review were managed using a shared document. Two reviewers independently screened eligible studies for selection. A third reviewer resolved any discrepancies that arose. A total of 33 articles were advanced from the Full Text Review stage for Data Extraction.

#### Data collection process/charting

Data from included studies were extracted using a systematic approach, wherein two team members independently extracted data then met to ensure consensus. Any discrepancies were resolved through reflexive group consensus, which consisted of a discussion and review by a third reviewer and the first author (JQ) to ensure accuracy. Themes were identified inductively by the first author (JQ) through iterative engagement with the charted data across all manuscripts, drawing on extracted wellbeing aspects, organizing categories, constructs, and model purposes and future directions. Candidate themes were generated by examining patterns of conceptual overlap and distinction across models. JQ then met with a co-author (Cerila Rapadas) to collaboratively and reflexively review, refine, and confirm the emerging thematic structure. The resulting seven themes were subsequently reviewed by the full Indigenous research team, whose collective knowledge of Turtle Island and Moananuiākea affirmed the cultural resonance and analytic integrity of the final thematic framework.

### Data extraction

Data extraction variables were determined by the JBI Manual for Evidence Synthesis and refined by piloting two randomly selected manuscripts. For each study, data were extracted to summarize contextual and methodological characteristics relevant to wellbeing frameworks. Information collected included the place and community context, and the type of work (e.g., development of a framework, model, or theory) conducted in the article or study. Participant information was recorded when available, including sample size and demographic characteristics. Methodological details were extracted, including study design and the specific data collection methods.

Definitions and terminology related to wellbeing (e.g., “wellbeing,” “quality of life,” or “wellness”) were summarized. The number and names of wellbeing organizing categories identified in each study were recorded. Features of each wellbeing framework were described using verbatim terminology from the source and categorized by overarching characteristics defined by the team. The research team also iteratively documented additional details or questions relevant to interpretation and synthesis.

### Synthesis of results

The synthesis process was designed to address the research questions and objectives by integrating and comparing a range of studies. Included studies were grouped according to their conceptual focus (e.g., type of framework, wellbeing domain, or population studied) to facilitate thematic and comparative analysis. Within each grouping, studies were summarized by setting, participant characteristics, study design, and key findings relevant to wellbeing.

Data were synthesized using a narrative approach, supported by tabular and visual summaries. Tables were used to present study characteristics, wellbeing definitions, organizing categories, and elements. A map was developed to illustrate locations of studies and compared to table contents. This combined narrative, tabular, and visual synthesis allowed for comprehensive mapping of the evidence, identification of conceptual overlaps and distinctions, and clarification of how wellbeing is defined and operationalized across the Indigenous populations of study.

## Results

### Literature selection

Database searches identified 2,966 records, comprising PubMed (*n* = 429), Web of Science (*n* = 1,011), EBSCO Academic Search Complete and CINAHL (*n* = 1,025), and ProQuest (*n* = 501). After removing 1,493 duplicates, 1,473 records were screened by title and abstract. Of these, 1,446 records were excluded, most commonly for not addressing Indigenous wellbeing, lacking a conceptual framework, or not focusing on wellness, wellbeing, or quality of life.

A total of 27 full-text manuscripts were sought from database searching, and all were successfully retrieved. Full-text screening resulted in 16 reports excluded, with reasons including: not addressing wellbeing/wellness/quality of life (*n* = 9), lacking a framework or model/theory (*n* = 5), focused solely on environmental wellbeing (*n* = 1), or a newer manuscript superseding the version retrieved (*n* = 1).

In addition to database searching, 89 records were identified through citation searching and professional networks (*n* = 47) and targeted Google Scholar searching (*n* = 42). Of these, 35 reports were retrieved for full-text screening. One report could not be retrieved, and 12 were excluded at full-text review due to: not addressing wellbeing/wellness/quality of life (*n* = 5), lacking an Indigenous wellbeing framework, model, or theory (*n* = 4), not focusing on Indigenous populations (*n* = 1), or a newer manuscript was located (*n* = 2). Across all sources, 33 articles met the inclusion criteria and were included in the final review.

### Characteristics of findings

The article citation, model name, discipline, publication, place and community, model or framework categorization (no theories were found), participant information, and study design are presented in Table 1. Table 2 describes aspects of Indigenous wellbeing from each model and framework, accompanied by Table 3 which provides total counts and percentages of these aspects. Verbatim organizing categories of each model and framework are outlined in Table 4. Descriptions of the model and framework purpose and future directions are provided in Table 5. Table 6 describes original definitions of wellbeing provided for each model or framework.

**Table 1:** Models and frameworks of wellbeing from Turtle Island and Moananuiākea.

Article citation	Model name	Discipline	Journal, book, or other	Moananuiākea, Turtle Island	Community and place	Type	Participants	Study design
Camacho et al. (74)	N/A	Interdisciplinary	Genealogy	Turtle Island	Queer and Trans Pacific Islanders (QTPI) (Puget Sound Area, Washington State, United States)	Model, Framework	Total: *n* = 12 Gender: 18.2% cisgender female, 9.1% cisgender male, 45.5% Fa’afafine, 9.1% Gela’, 9.1% Tinalao’an, 9.1% not quantifiable Role: Queer and Trans Pacific Islander community members	Qualitative (including storytelling), Indigenist Collaborative Research
Johnson et al. (143)^†^	The Ngaruroro model of Māori wellbeing	Transdisciplinary	International Journal of Environmental Research and Public Health	Moananuiākea	Māori Aotearoa (New Zealand)	Model	Total: *n* = 24 Gender: 50% women, 50% men Role: Māori community members	Qualitative (including storytelling), Kaupapa Māori Methodology
Shea (76)	The Living Well Model	N/A	Aacimotaatiiyankwi (blog)	Turtle Island	Myaamiaki; Miami Nation (Oklahoma and Indiana, United States)	Model	N/A	N/A
Paul et al. (77)	The Piikani Well-being Index (PWI)	Geography	Environment and Planning F: Philosophy, Theory, Models, Methods and Practice	Turtle Island	Amskapi Piikani (Blackfeet Nation) (Montana, United States)	Model	Total: *n* = 20 Role: Blackfeet planning practitioners, community organizers, thought leaders, traditional knowledge holders, and decision makers	Survey, qualitative (including storytelling), quantitative (statistical and GIS analysis), community-based
Fletcher et al. (50)	The IQI model of health	Public Health	Canadian Journal of Public Health	Turtle Island	Inuit Peoples (Nunavik, Quebec, Canada)	Model	Total: *n* = 85+ Gender: “A diversity of men, women…” Role: Inuit community members (youth and elders)	Document review, qualitative (including storytelling), community-based participatory research
Smith et al. (67)	The Good Spirit, Good Life tool and framework	Gerontology	The Gerontologist	Moananuiākea	Aboriginal Peoples (Perth and Melbourne, Australia)	Framework	Total: *n* = 69 Gender: 77% women Role: Elder Aboriginal Australians, largely recipients of aged care	Qualitative (including storytelling), participatory action research
Kanaʻiaupuni et al. (70)	The Pua Model: A Native Hawaiian perspective on well-being	Education	KA HUAKA‘I Native Hawaiian Educational Assessment 2021	Moananuiākea	Kānaka Maoli (Hawai‘i)	Model	Role: Native Hawaiian families and communities	Document review
Dion et al. (53)	N/A	Psychiatry	Transcultural Psychiatry	Turtle Island	Inuit Peoples (Nunavik, Quebec, Canada)	N/A	Total: *n* = 19 Role: Inuit key-informants involved in community wellness work	Qualitative (including storytelling), participatory research
Panapa et al. (55)	The Ola Lei Framework	Interdisciplinary	Journal of the Polynesian Society	Moananuiākea	Tuvaluan Peoples (Funafuti and Vaitupu, Tuvalu and Aotearoa (New Zealand)	Framework	Role: Community elders, leaders, health professionals, traditional healers, schoolteachers, students, and community members	Observational, qualitative (including storytelling), ethnographic research
Cardinal and Pepler (52)	The Community Journey of Change model	Policy	The International Indigenous Policy Journal	Turtle Island	First Nations and Inuit Peoples (Saskatchewan, Ontario, Nunavut, and British Columbia, Canada)	Model	Role: First Nations and Inuit communities who participated in walking the prevention pathway (violence prevention) research	Survey, document review, qualitative (including storytelling)
Garvey et al. (64)	The Fabric of Aboriginal and Torres Strait Islander Wellbeing model	Transdisciplinary	International Journal of Environmental Research and Public Health	Moananuiākea	Aboriginal and Torres Strait Islander Peoples (Australia)	Model	Total: *n* = 359 Gender: 57.1% female, 42.9% male Role: Aboriginal and Torres Strait Islander community members	Qualitative (including storytelling), Indigenist Research
Odom et al. (73)	Pilinahā: The Four Connections Framework	Nutrition	Current Developments in Nutrition	Moananuiākea	Native Hawaiians and Kōkua Kalihi Valley community members (Kalihi, O‘ahu, Hawai‘i	Framework	Total: *n* = 38 Gender: 65.8% women, 34.2% men Role: Native Hawaiians or individuals who worked with Native Hawaiian communities; Farmers, producers, educators, academic professors, and leaders of cultural/health/youth organizations	Qualitative (including storytelling), Indigenous Research
Kyoon-Achan et al. (48)	First Nations Mental Wellness Framework	Interdisciplinary	International Journal of Culture and Mental Health	Turtle Island	First Nations Peoples (Manitoba, Canada)	Framework	Total: *n* = 61 Role: First Nations leaders and elders	Qualitative (including storytelling), community-based participatory research, strengths-based approach
Kūkulu Kumuhana Planning Committee (69)	Kūkulu Kumuhana	Interdisciplinary	Kūkulu Kumuhana: Creating Radical and New Knowledge to Improve Native Hawaiian Wellbeing	Moananuiākea	Native Hawaiians (Hawai‘i)	Framework	Total: *n* = 49 Role: Native Hawaiian leaders from multiple disciplines (education, health, art, social work, psychology, etc.)	N/A
Wong-Wilson (71)	Wong-Wilson’s New Native Hawaiian Well-Being Model	Education	Thesis	Moananuiākea	Native Hawaiians; Hawai‘i Community College Students and Faculty (Hilo, Hawai‘i Island, Hawai‘i)	Model	Total: *n* = 534+ Role: Hawai‘i Community College students and faculty, some Native Hawaiian	Document Review, Survey, Qualitative (including storytelling), Indigenous Research
Cairney et al. (66) Cairney and Abbott (80)*	The Interplay Wellbeing Framework	Economics	Cooperative Research Centre for Remote Economic Participation Working Paper CW024	Moananuiākea	Aboriginal and Torres Strait Islander Peoples (Remote Australia)	Framework	Total: *n* = 846–1,046 Role: Aboriginal and Torres Strait Islander young adult researchers, community leaders, and respected elders	Mixed methods: quantitative, qualitative, document review, case study, participatory action research, community-based participatory research
Ontario’s Aboriginal Health Access Centres (51)	AHAC Model of Wholistic Health and Wellbeing	Health	AHAC Model of Wholistic Health and Wellbeing: A Time for Reconciliation	Turtle Island	Aboriginal Peoples (Ontario, Canada)	Model	N/A	N/A
Ministry of Health Ontario (49)	First Nations Mental Wellness Continuum Model	Health	First Nations Mental Wellness Continuum Framework	Turtle Island	First Nations Peoples (Canada)	Framework	Role: First Nations community members, the First Nations and Inuit Health Branch (FNIHB) of Health Canada, the Assembly of First Nations (AFN), and Indigenous mental health leaders from various First Nations non-governmental organizations	N/A
Gee et al. (65)	Social and Emotional Wellbeing from an Aboriginal and Torres Strait Islanders’ Perspective	Psychology	Working Together: Aboriginal and Torres Strait Islander Mental Health and Wellbeing Principles and Practices	Moananuiākea	Aboriginal and Torres Strait Islander Peoples (Australia)	Framework	Total: *n* = 300+ Role: Attendees/members at national and state social and emotional wellbeing conferences; community consultants	N/A
Te Moananui-Makirere et al. (61)	The Te Ara Whakapikiōranga Framework	Social Work	Aotearoa New Zealand Social Work	Moananuiākea	Māori Aotearoa (New Zealand)	Framework	Total: *n* = 1,039+ Role: Representatives from Whānau Ora regional leadership groups, Whānau Ora Collective service providers, marae representatives, Māori Women’s Welfare League, government agencies, and whānau themselves	Observational, qualitative (including storytelling), Whānau-centred research
Kingsley et al. (63)	Exploratory Framework for Aboriginal Victorian peoples’ wellbeing	Transdisciplinary	International Journal of Environmental Research and Public Health	Moananuiākea	Aboriginal Peoples (Victoria, Australia)	Framework	Total: *n* = 3 Role: Traditional custodian groups and Aboriginal land managers in Victoria	Qualitative (including storytelling), document review
Drabek (75)	Qik’rtarmiut Sugpiat’stun Sug’ucirpet: The Kodiak Alutiiq People’s Way of Being Human	Education	Thesis	Turtle Island	Alutiiq (Sugpiaq) (Kodiak, Alaska, United States)	Model	N/A	Document review
New South Wales Government (62) New South Wales Government (79)*	The Wellbeing Framework	Interdisciplinary	Strengthening Aboriginal Community Wellbeing Toolkit: User Manual	Moananuiākea	Aboriginal Peoples (Wellington and La Perouse, Australia)	Framework	N/A	N/A
Mark and Lyons (59)	Te Whetu	Social Science	Social Science and Medicine	Moananuiākea	Māori Aotearoa (New Zealand)	Model	Total: *n* = 6 Gender: 83.3% female, 16.7% male Role: Māori spiritual healers	Qualitative (including storytelling)
Australian Bureau of Statistics (68)	The Indigenous Wellbeing Framework	Statistics	Australian Bureau of Statistics	Moananuiākea	Aboriginal and Torres Strait Islander Peoples (Australia)	Framework	Role: Indigenous researchers and other stakeholders	N/A
Murray (60)	Te Punga Oranga	Nursing	Tau Ora for Our People	Moananuiākea	Māori Aotearoa (New Zealand)	Model	N/A	N/A
McGregor et al. (1, 78)	Ho‘oulu Lāhui Aloha ‘Raising a Beloved Nation’ Ecological Model of Native Hawaiian Wellbeing	Medical and Public Health	Pacific Health Dialog	Moananuiākea	Native Hawaiians (Hawai‘i)	Model, framework	N/A	N/A
Durie (58)	Te Pae Māhutonga	Health	Health Promotion Forum of New Zealand Newsletter	Moananuiākea	Māori Aotearoa (New Zealand)	Model	N/A	N/A
van Uchelen et al. (54)	N/A	Interdisciplinary	Canadian Journal of Community Mental Health	Turtle Island	First Nations People (Downtown Eastside, Vancouver, Canada)	Framework	Total: *n* = 31 Gender: 51.6% female, 48.4% male Role: Aboriginal peoples living and/or working in Vancouver’s Downtown Eastside area	Qualitative (including storytelling)
Rezentes (72)	The Lōkahi Triangle	Psychology	Ka Lama Kukui Hawaiian Psychology: An Introduction	Moananuiākea	Hawaiians (Hawai‘i)	Model	N/A	N/A
Ministry of Health Ontario (49)	An Aboriginal Framework for Wholistic Health and Well-Being	Health	New Directions: Aboriginal Health Policy for Ontario	Turtle Island	First Nation and Aboriginal Peoples (Ontario, Canada)	Framework	Total: *n* = 7,000+ Role: Aboriginal community members	N/A
Pere (57)	Te Wheke	Education	Te Wheke: a celebration of infinite wisdom	Moananuiākea	Māori Aotearoa (New Zealand)	Model	N/A	N/A
Durie (20)	Te Whare Tapa Whā^‡^	Social Science	Social Science and Medicine	Moananuiākea	Māori Aotearoa (New Zealand)	Model	N/A	N/A

*For models and frameworks with more than one manuscript, data is presented here from the more recent manuscript. Where manuscripts were published in the same year, authors were contacted to determine manuscript for data extraction.

†Author provided additional information.

‡The name, though absent from the reviewed manuscript, was found in subsequent publications.

**Table 2:** Aspects of Indigenous wellbeing.

Article citation	Model name	Holism	Relational(ity)	(Inter)connection	Ecological	Physical	Psychological/mental	Collectivist	Time as cyclical	Intergenerational	Family	Community	Personal sovereignty	Cultural sovereignty	Political sovereignty	Economic sovereignty	Land stewardship	Land as family	Balance	Reciprocity	Culture	Stories	Spirituality	Material quality of life	Supportive determinants	Detrimental determinants	Outcomes	Hedonic	Eudaimonic
Camacho et al. (74)	N/A	X	X	X	X	X	X	X	X	X	X	X	X	X	X		X		X	X	X	X	X	X	X	X		X	
Johnson et al. (143)	The Ngaruroro model of Māori wellbeing	X	X	X		X	X	X		X	X	X	X	X	X	X	X	X	X	X	X	X	X		X	X	X	X	
Shea H. (76)	The Living Well Model	X	X	X	X	X	X	X		X	X	X			X		X			X	X	X	X		X			X	X
Paul et al. (77)	The Piikani Well-being Index (PWI)	X	X	X		X		X				X			X		X	X		X	X			X	X	X		X	
Fletcher et al. (50)	The IQI model of health	X	X	X	X	X	X	X		X	X	X	X	X			X		X	X	X	X	X	X	X	X		X	X
Smith et al. (67)	The Good Spirit, Good Life tool and framework	X	X	X	X	X	X	X		X	X	X	X	X	X				X	X	X	X	X	X	X	X		X	X
Kanaʻiaupuni et al. (70)	The Pua Model: A Native Hawaiian perspective on well-being	X	X	X		X	X	X	X	X		X	X	X	X	X	X		X	X	X		X	X	X	X			X
Dion et al. (53)	N/A		X	X			X	X		X	X	X	X	X				X			X				X	X			
Panapa et al. (55)	The Ola Lei Framework		X	X		X	X	X		X	X	X									X		X	X				X	
Cardinal and Pepler (52)	The Community Journey of Change model	X	X	X	X	X	X		X		X	X	X	X			X	X	X		X	X	X		X	X	X		X
Garvey et al. (64)	The Fabric of Aboriginal and Torres Strait Islander Wellbeing model	X	X	X	X	X	X	X			X	X	X	X		X			X	X	X	X		X	X	X	X		
Odom et al. (73)	Pilinahā: The Four Connections Framework	X	X					X	X	X		X					X	X	X	X	X		X						
Kyoon-Achan et al. (48)	First Nations Mental Wellness Framework	X	X					X		X	X	X					X	X	X		X	X	X		X				X
Kūkulu Kumuhana Planning Committee (69)	Kūkulu Kumuhana	X	X	X	X			X		X	X	X	X	X	X		X	X	X	X	X	X	X	X	X				X
Wong-Wilson (71)	Wong-Wilson’s New Native Hawaiian Well-Being Model	X	X	X		X	X	X		X	X	X	X	X		X		X	X	X	X	X	X	X					
Cairney et al. (66) Cairney and Abbott (80)*	The Interplay Wellbeing Framework	X	X	X	X		X				X	X	X	X	X		X	X	X		X	X	X		X		X		
Ontario’s Aboriginal Health Access Centres (51)	AHAC Model of Wholistic Health and Wellbeing	X	X	X	X	X	X	X	X	X		X	X	X	X	X	X			X	X	X	X		X			X	X
Ministry of Health (49)	First Nations Mental Wellness Continuum Model	X	X	X	X	X	X	X	X	X	X	X	X	X	X	X	X	X	X	X	X	X	X	X	X	X	X		
Gee et al. (65)	Social and Emotional Wellbeing from an Aboriginal and Torres Strait Islanders’ Perspective	X	X	X	X	X	X	X		X	X	X	X	X	X	X	X		X	X	X	X	X	X	X	X		X	X
Te Moananui-Makirere et al. (61)	The Te Ara Whakapikiōranga Framework	X	X	X	X	X	X	X		X	X	X	X	X	X	X	X	X	X	X	X	X	X		X	X	X		
Kingsley et al. (63)	Exploratory Framework for Aboriginal Victorian peoples’ wellbeing	X	X	X	X			X			X						X	X	X	X	X	X	X		X	X			
Drabek (75)	Qik’rtarmiut Sugpiat’stun Sug’ucirpet: The Kodiak Alutiiq People’s Way of Being Human	X	X	X		X					X									X	X		X					X	
New South Wales Government (62) New South Wales Government (79)*	The Wellbeing Framework	X	X	X	X	X	X	X		X	X	X		X	X		X	X		X	X	X		X	X	X		X	X
Mark and Lyons (59)	Te Whetu	X	X	X	X	X	X	X		X	X	X		X	X		X	X	X		X	X	X		X	X		X	X
Australian Bureau of Statistics (68)	The Indigenous Wellbeing Framework	X	X			X	X				X	X		X	X	X					X			X	X	X			
Murray (60)	Te Punga Oranga	X	X	X	X	X	X	X		X	X	X	X	X		X	X	X	X	X	X		X	X	X	X	X	X	
McGregor et al. (1, 78)	Ho‘oulu Lāhui Aloha ‘Raising a Beloved Nation’ Ecological Model of Native Hawaiian Wellbeing	X	X	X	X	X	X	X	X	X	X	X	X	X	X	X	X	X	X	X	X	X	X	X	X	X	X	X	X
Durie (58)	Te Pae Māhutonga	X	X	X	X	X		X		X		X	X	X	X	X	X	X	X	X	X	X	X		X	X	X		
van Uchelen et al. (54)	N/A	X	X	X		X	X	X		X	X	X							X	X	X	X	X		X	X	X	X	X
Rezentes (72)	The Lōkahi Triangle	X	X	X			X	X		X	X								X		X		X						X
Ministry of Health Ontario (49)	An Aboriginal Framework for Wholistic Health and Well-Being	X	X	X		X	X	X		X	X	X	X						X		X		X		X	X			
Pere (57)	Te Wheke	X	X	X		X	X	X	X	X	X	X						X	X	X	X	X	X	X	X			X	
Durie (20)	Te Whare Tapa Whā^‡^	X	X	X	X	X	X	X		X		X	X	X			X	X	X	X	X	X	X		X		X		

*For models and frameworks with more than one manuscript, data is presented here from the more recent manuscript. Where manuscripts were published in the same year, authors were contacted to determine manuscript for data extraction.

‡The name, though absent from the reviewed manuscript, was found in subsequent publications.

**Table 3:** Total counts and percentages for aspects of Indigenous wellbeing.

Aspect of Indigenous wellbeing	Frequency	Percentage
Relational(ality)	33	100%
Culture	33	100%
Holism	31	94%
(Inter)connection	30	91%
Community	30	91%
Collectivist	29	88%
Spirituality	28	85%
Supportive determinants	28	85%
Intergenerational	27	82%
Physical	26	79%
Psychological/mental	26	79%
Family	26	79%
Balance	25	76%
Reciprocity	24	73%
Stories	23	70%
Cultural sovereignty	22	67%
Land stewardship	22	67%
Detrimental determinants	21	64%
Personal sovereignty	20	61%
Ecological	19	58%
Land as family	19	58%
Political sovereignty	17	52%
Material quality of life	17	52%
Hedonic	16	48%
Eudaimonic	14	42%
Economic sovereignty	12	36%
Outcomes	11	33%
Time as cyclical	8	24%

**Table 4:** Organizing categories from models and frameworks.

Article citation	Model name	Imagery used	Organizing category 1	Organizing category 2
Camacho et al. (74)	N/A	Clay pot with shattered fragments, sources of health and well-being depicted as reparative resin	Clay Pot Piece 1: Family Clay Pot Piece 2: Community Clay Pot Piece 3: Culture	Components: Mechanism that restores (Indigenous connectedness with values); Values (relational ways of being, inágofli’e’ and alofa); Safe spaces (chosen family, community care, diaspora); colonial disruptions (policing of QTPI identity, invisibility and erasure, violence)
Johnson et al. (143)	The Ngaruroro model of Māori wellbeing	Streams of water	Theme 1: Here Tāngata (connection to social and familial ties) Theme 2: Tinana (lifestyle choices related to the tinana and physical health) Theme 3: Ngākau (capacities related to the inner-world) Theme 4: Wairua (lifestyle choices related to spirit and interconnectedness) Theme 5: Taiao (connection to the environment) Theme 6: Matea (capacities to meet core needs) Theme 7: Mana (capacities related to exercising mana) Theme 8: Taonga Tuku Iho (connection to cultural treasures)	Here Tāngata Items: Whānau (family); Hapū (sub-tribe); Iwi (tribe); Tīpuna (ancestors); Hoa (friends); Hapori (communities) Tinana Items: Kai (diet); Kori tinana (physical activity); Moe (sleep); Kai whakapiri (substances that people use to feel a sense of connection or self-medicate); Kanohi kitea (having your face physically seen) Ngākau Items: Kare-ā-roto (emotions); Whakaaro thoughts); Waiaro (attitudes); Aroha (love); Pāmamae (trauma, grief, deep pain) Wairua Items: Atua [Māori deities, ancestors of continued influence, god(s)]; Wana (exhilarating and breath-taking experiences); Wāhi wairua (spaces that nurture your sense of wairua); Mahi aroha (activities or work you do for passion, love, or service); Poipoi i te mauri (nurturing the lifeforce of the beings, spaces, and things around you) Taiao Items: Whenua (land); Wai tai (bodies of salt water); Wai māori (bodies of fresh water); Ngahere (bush and forests); Ngā rangi (celestial bodies) Matea Items: Whai mātauranga (acquiring knowledge); Tuku mātauranga (passing on knowledge); Kainga (housing); Pūtea (money); Wā whakatā (relaxation) Mana Items: Tū tangata (express and stand in the fullness of your identity); Whiriwhiri (make key decisions about how your life unfolds); Manaaki (uplift, take care of, and/or be hospitable to others); Whakatere (navigate challenges in life); Tū toa (stand accomplished in a skill or area) Taonga Tuku Iho Items: Te reo Māori (the Māori language); Mātauranga Māori (traditional and contemporary Māori knowledge); Tikanga Māori (Māori customs, protocols, and ways of being); Uaratanga Māori (Māori values); Tūrangawaewae (traditional and contemporary places of belonging)
Shea (76)^†^	The Living Well Model	Mankiišaahkwi (sassafras) leaves and face	Tenet 1: pilakioni ‘health’ Tenet 2: aweentioni ‘peace’	Knowledge Competencies: Akimaayoni “Civic Knowledge”; Nakaaniaki “Ancestral/Historical Knowledge”; Myaamiaataweenki “Language competence”; Myaamia Nipwaayoni “Cultural competence”; Ašiihkiwi kiišikwi “Ecological Competence” Values: neepwaahkaayankwi ‘we are wise, conscious, aware’; eeyaakwaamisiyankwi ‘we strive for (something)’; eeweentiiyankwi ‘we are related to each other’; peehkinaakosiyankwi ‘we are generous, kind’; aahkohkeelintiiyankwi ‘we care for each other’; neehweeyankwi ‘we speak well’; paahpilweeyankwi ‘we joke, are humorous’; aahkwaapawaayankwi ‘we dream’ Intentional Interactions: awiiyoome ‘body’; mihtohseeniaki ‘the people’; manetawioni ‘spirit’; išiteehioni ‘thought’; Myaamionki ‘Myaamia places’
Paul et al. (77)	The Piikani Well-being Index (PWI)	N/A	Indicator Area 1: Sokinaapi or Saam (our health) Indicator Area 2: Awahsin (where our food comes from, our food sovereignty) Indicator Area 3: Niitsítapia’pii (our culture) Indicator Area 4: Niipáítapiiwahsin Sopoksistotsi (social and educational, life-way) Indicator Area 5: Kitawahsinnooni/Otoi’tsikat Our Lands and Stewardship (to care for, biosystem stewardship) Indicator Area 6: Aókakihtsimaa (institutional capacity and traditional governance) Indicator Area 7: Iksiststsoohsi (our business, affairs and economics) Indicator Area 8: Kiistónnoon Inaanatoo (our land tenure)	Core cultural values active in Amskapi Piikani community development, educational, and governance systems: Tsi-ksi-ka-ta-pi-wa-tsin (blackfeet way of knowing); Nin-na-wa-tsin (being a leader); Ini-yimm (respect); Ni-ta-pi-pa-ta-pi-tsin (living in a good way); Ii-yi-kah-kii-ma-tsin (trying hard); Aoh-kan-otah-tomo (accepting everyone); Ii-ta-mii-pa-ta-pi-yoip (happy living)
Fletcher et al. (50)	The IQI model of health	Flower and soil	Dimension 1: Ilusirsusiarniq (bodily health) Dimension 2: Qanuinngisiarniq (broad sense of well-being) Dimension 3: Inuuqatigiitsianiq (ideal form of social well-being)	Community determinants of health: food, community, family, land, economy, identity, knowledge, services
Smith et al. (67)	The Good Spirit, Good Life tool and framework	Sun	Necessity 1: Promote healing Necessity 2: Strengthen Necessity 3: Overcome barriers Necessity 4: Protect	Factors: Family and friends; country; community; culture; health; respect; elder role; supports and services; safety and security; spirituality; future planning; basic needs
Kanaʻiaupuni et al. (70)	The Pua Model: A Native Hawaiian perspective on well-being	Pua (flower)	Dimension 1: Social Dimension 2: Physical Dimension 3: Educational Dimension 4: Material and Economic Dimension 5: Spiritual and Emotional	Social: ‘Ohana/Lāhui; ‘Ōlelo Hawai’i; Engaged citizens; safe communities Physical: Ola kino/Ola pono; health/nutrition; Longevity; Aloha ‘āina Educational: ‘Ike kupuna; Hawaiian culture-based education; knowledge/intellect; innovation Material and economic: Waiwai/Lawa pono; income/employment; homeownership; ‘Āina mole Spiritual AND emotional: Mana/Pono; Hawaiian identity; sense of place; mental health
Dion et al. (53)	N/A	Venn diagram	Principle 1: Support individual and collective self-determination Principle 2: Enhance individual and collective strength and confidence Principle 3: Focus on the interconnection between family and community members, and the land	Approaches: Traditional and cultural approaches Mentorship and consultations with elders; group support and healing; community activities and resources; explicit education-oriented prevention and promotion activities
Panapa et al. (55)	The Ola Lei Framework	Octopus	Central Quality 1: Filemuu ‘harmoniousness, peacefulness’ Central Quality 2: Iaia ‘happiness, contentment’ Central Quality 3: Malosi ‘fitness’ Central Quality 4: Ola leva ‘longevity’	Practices and pragmatic qualities: Meakai e lava e lei (food abundance and quality); Tuu-maa (cleanliness); Toka (readiness); Galue malosi (hard work); Maumea or Maukoloa (wealth); Poto faka-Tuvalu or Logo (traditional skills and knowledge); Talitonu e Fakatuanaki ki te Atua (belief and faith in god); Lei a te masaki (recovery from illness or disease)
Cardinal and Pepler (52)	The Community Journey of Change model	Circular diagram	Relational determinants of health Cycle of violence Circle of wellness	Foundations: physical; spiritual; mental; emotional
Garvey et al. (64)	The Fabric of Aboriginal and Torres Strait Islander Wellbeing model	Woven fabric	Aspects of Aboriginal and Torres Strait Islander life 1: Family Aspects of Aboriginal and Torres Strait Islander life 2: Community Aspects of Aboriginal and Torres Strait Islander life 3: Culture	Foundations of wellbeing: belonging and connection; holistic health; purpose and control; dignity and respect; basic needs
Odom et al. (73)	Pilinahā: The Four Connections Framework	Hoaka (crescent moon/bowl)	Connection 1: Connection to place: To have a kinship with ‘āina (land) Connection 2: Connection to community: To love and be loved; to understand and be understood Connection 3: Connection to past and future: To have kuleana (a purpose in the world) Connection 4: Connection to your better self: To find and know yourself	N/A
Kyoon-Achan et al. (48)	First Nations Mental Wellness Framework	Nested Medicine Wheel	Medicine wheel: Quadrant 1: Spiritual Quadrant 2: Emotional Quadrant 3: Mental Quadrant 4: Physical	Themes: balance and connection; community; elders and family; social contribution; meaning and purpose; spirituality; connection to the land; history and stories; traditional teachings; language; belonging; identity; stability
Kūkulu Kumuhana Planning Committee (69)	Kūkulu Kumuhana	Circular diagram§	Level 1: Family Level 2: Community Level 3: Organization Level 4: Policy	Dimensions: Ea (self-determination); ‘Āina Momona (healthy and productive land and people); Pilina (mutually sustaining relationships); Waiwai (ancestral abundance); ‘Ōiwi (cultural identity and native intelligence); Ke Akua Mana (spirituality and sacredness of mana)
Wong-Wilson (71)	Wong-Wilson’s New Native Hawaiian Well-Being Model	Nested circular diagram	Centre of being: Kanaka | ‘Āina Sphere 1: Ohana | Lāhui Sphere 2: Kaiaulu Sphere 3: Lāhui | Ohana Framework: Kanaka | ‘Āina	Social; material and economic; knowledge; physical and emotional Kuleana (responsibilities); Pono (harmony); Nā ʻaumakua (ancestors) and akua (Gods)
Cairney et al. (66) Cairney and Abbott (80)*	The Interplay Wellbeing Framework	Radial diagram	Domain 1: Culture Domain 2: Empowerment Domain 3: Community Domain 4: Education Domain 5: Employment Domain 6: Health	Culture experienced as: language; kinship; law; ceremony; land
Ontario’s Aboriginal Health Access Centres (51)	AHAC Model of Wholistic Health and Wellbeing	Medicine Wheel	Element 1: Culture, our ways of knowing and being Element 2: North (spiritual, reclamation, generosity) Element 3: South (learning, language, mental) Element 4: East (emotional, healing, belonging) Element 5: West (physical, teaching, interdependence)	N/A
Ministry of Health (49)	First Nations Mental Wellness Continuum Model	Nested Medicine Wheel	Four Directions-Outcomes 1: Hope Four Directions-Outcomes 2: Belonging Four Directions-Outcomes 3: Meaning Four Directions-Outcomes 4: Purpose	Sections: community-kinship, clan elders and community; populations; specific population needs; continuum of essential services; supporting elements; partners in implementation; Indigenous social determinants of health; key themes for mental wellness; culture as foundation
Gee et al. (65)	Social and Emotional Wellbeing from an Aboriginal and Torres Strait Islanders’ Perspective	Nested circular diagram	Domain 1: Connection to spirit, spirituality and ancestors Domain 2: Connection to body Domain 3: Connection to mind and emotions Domain 4: Connection to family and kinship Domain 5: Connection to community Domain 6: Connection to culture Domain 7: Connection to country	Expressions Experiences
Te Moananui-Makirere et al. (61)	The Te Ara Whakapikiōranga Framework	N/A	Wāhanga 1: Te Āu I Te Whānau (the self in the family) Wāhanga 2: Puna Ki Te Puna (practice wisdom) Wāhanga 3: Te Tohu O Te Rangatira (Whānau-centered leadership) Wāhanga 4: Hono Mai Hono Atu (connections and relationships)	Te Āu I Te Whānau (the self in the family) Ātua; Whakapapa (genealogy/lineage); Whare wānanga Puna Ki Te Puna (practice wisdom) Wāhanga: MataPuna (the face or foundation of practice wisdom); TūPuna (the stance or history of practice wisdom); MokoPuna (the imprint of practice wisdom) Te Tohu O Te Rangatira (Whānau-centered leadership) Wāhanga: Te kai ā te rangatira, he kōrero (communication that sustains); Te mahi a te rangatira, hei whakatira i te whānau, hapu, iwi (leadership that strengthens whānau, hapū and iwi); Te tohu o te rangatira hei whakapiki i te oranga whānau (whānau lead their own wellbeing) Hono Mai Hono Atu (Connections and relationships): Hono Mai (identification of whānau-centered principles and beliefs); Hono Atu (application of the whakapikioranga framework)
Kingsley et al. (63)	Exploratory Framework for Aboriginal Victorian peoples’ wellbeing	Tree and soil	Aboriginal forces impacting on wellbeing Western/downward forces	Aboriginal forces impacting on wellbeing: roots (biology; learnt experiences; built environment; country; partnerships); branches (traditional knowledge; ancestry; caring for country; consultation, respect, consistency, and employment opportunities; missions, urban spaces); western/downward forces (politics; health issues; loss of traditional cultural knowledge; racism; native title; colonisation; destruction and recognition of country)
Drabek (75)	Qik’rtarmiut Sugpiat’stun Sug’ucirpet: The Kodiak Alutiiq People’s Way of Being Human	Llam Sua icon	Sphere 1: Nuna – place: physical sphere Sphere 2: Suuget – people: social sphere Sphere 3: Keneq – fire, process: cognitive sphere Sphere 4: Anerneq – breath, spirit: spiritual sphere Sphere 5: Lla – conscience: ethical sphere	Nuna/place: physical sphere (Values: Nunapet - ties to our homeland; Nunapet Carliarluki - Stewardship of animals, land, sky, and waters; Unguwacirpet - A subsistence lifestyle respectful and sustained by the natural world) Suuget/people: social sphere [values: Suupet - Our people (community): we are responsible for each other and ourselves; Cuqllipet - our elders; Ilaapet - our family and kinship of ancestors and living relatives] Keneq/fire; process: cognitive sphere (values: Sugt’stun Niuwacipet, Yugnerpet - our heritage language; Liicukukut - learning by doing, observing and listening; Piciipet Uswituu’uq - traditional arts, skills, and ingenuity) Anerneq/breath; spirit: spiritual sphere (Values: Agavumaukut - faith and spiritual life from ancestral beliefs to the diverse faiths of today); Englarstaisgnukut – humor Lla/conscience: ethical sphere (values: Ilakuisngukut - Sharing: we welcome everyone; Sugtanartukut, Uqwarnartukut - trust; Ling’aklluki - respect for self, others and the environment is inherent in all values)
New South Wales Government (62) New South Wales Government (79)*	The Wellbeing Framework	Circles/radial diagram	Characteristic 1: Access to country Characteristic 2: Community health and safety Characteristic 3: Cultural identity Characteristic 4: Economic strength and development Characteristic 5: Education and learning Characteristic 6: Infrastructure and services Characteristic 7: Leadership, empowerment and influence Characteristic 8: Sense of community	N/A
Mark and Lyons (59)	Te Whetu	Star	Aspect 1: Hinengaro – mind Aspect 2: Tinana – body Aspect 3: Whenua – land Aspect 4: Whānau/Whakapapa - family and genealogy Aspect 5: Wairua - spirit	Themes: Impact of colonisation; Wairuatanga (spirituality); Whānau and Whakapapa (family and genealogy); Whenua (land); Māori healing techniques (Rongoā)
Australian Bureau of Statistics (68)	The Indigenous Wellbeing Framework	Nested circular diagram	Domain 1: Culture, heritage and leisure Domain 2: Family, kinship and community Domain 3: Health Domain 4: Education, learning and skills Domain 5: Customary, voluntary and paid work Domain 6: Income and economic resources Domain 7: Housing, infrastructure and services Domain 8: Law and justice Domain 9: Citizenship and governance	Individual level: social, cultural, and economic environments
Murray (60)	Te Punga Oranga	Fern	Rau 1: Pāpori (social) Rau 2: Taiao (environmental) Rau 3: Mahi (occupational) Rau 4: Tinana (physical) Rau 5: Aronganui (emotional) Rau 6: Whānau and Iwi (family and community) Rau 7: Hinengaro (Mental) Rau 8: Wairua (Spiritual)	N/A
McGregor et al. (1, 78)	Ho‘oulu Lāhui Aloha ‘Raising a Beloved Nation’ Ecological Model of Native Hawaiian Wellbeing	Nested circular diagram and tree map	Dimension 1: ‘Āina wellbeing Dimension 2: Nation wellbeing Dimension 3: Community wellbeing Dimension 4: Ohana wellbeing Dimension 5: Individual wellbeing	‘Āina wellbeing components: water; native flora and fauna, stewardship; natural elements as interconnected and interdependent; wai is the most important natural element to sustain life; ancestral place names/chants/legends transmit place-based knowledge; importance of Malama ‘Āina and Lōkahi between humans and nature Nation wellbeing components: historically constituted stable community; language, culture and spirituality; economic life; national land base; political sovereignty, governance, Hawaiian rights, access rights, land rights Community wellbeing components: integrity of Ahupua‘a (watershed), Moku (district), Moku‘aina (island); informal networking and sharing of support and interest; cultural, spiritual and social places to gather/provide services/hold activities; economic development; leadership and organization (formal) ʻOhana level components: Piko ‘aumakua (ancestors); Piko ‘iewe (immediate family); Piko ‘iwi kuamo‘o (future)
Durie (58)	Te Pae Māhutonga	Southern Cross constellation§	Key tasks of health promotion 1: Mauriora (access to te ao Māori) Key tasks of health promotion 1: 2: Waiora (environmental protection) Key tasks of health promotion 1: 3: Toiora (healthy lifestyles) Key tasks of health promotion 1: 4: Te Oranga (participation in society) Pointer 1: Nga Manukura (leadership) Pointer 2: Te Mana Whakahaere (autonomy)	Mauriora tasks: access to language and knowledge; access to culture and cultural institutions; access to Māori economic resources; to social resources; to societal domains Waiora Outcomes: water free from pollutants; clean air; earth abundant in vegetation; healthy noise levels; opportunities to experience natural environment Toiora areas for consideration: harm minimization; targeted interventions; risk management; cultural relevance; positive development Te Oranga participation in: economy; education; employment; the knowledge society; decision making Nga Manukura reflects: community leadership; health leadership; tribal leadership; communication; alliances between leaders and groups Te Mana Whakahaere evidence: control; recognition of group aspirations; relevant processes; sensible measures and indicators; capacity for self-governance
van Uchelen et al. (54)	N/A	N/A	Theme 1: Sense of community Theme 2: Identity Theme 3: Traditions Theme 4: Contribution Theme 5: Spirituality Theme 6: Living in a good way Theme 7: Coming through hardship Theme 8: Illness	N/A
Rezentes (72)	The Lōkahi Triangle	Triangle	Dimension 1: Ke Akua - god(s) Dimension 2: ‘Āina – nature Dimension 3: Kanaka - mankind	‘Āina - nature sub-domains: physical ‘Āina; psychological ‘Āina; spiritual ‘Āina
Ministry of Health Ontario (49)	An Aboriginal Framework for Wholistic Health and Well-Being	Nested circular diagram	Stages of life 1: Infants and children Stages of life 2: Youth Stages of life 3: Adults Stages of life 4: Elderly	Needs of the individual, family and community: spiritual; physical; emotional; mental continuum of care (promotion, prevention, curative, rehabilitation)
Pere (57)	Te Wheke	Octopus	Head: child/family Tentacle: dimensions Suckers: Facets within each dimension	Dimension 1: Mauri - life force in people and objects Dimension 2: Mana ake - unique identity of individuals and family Dimension 3: Taha Tinana - physical wellbeing Dimension 4: Whanaungatanga - extended family Dimension 5: Hinengaro - the mind Dimension 6: Wairuatanga – spirituality Dimension 7: Hā a koro ma, a kui ma - breath of life from forbears Dimension 8: Whatumanawa - the open and health expression of emotion
Durie (20)	Te Whare Tapa Whā^‡^	Four-walled house §	Dimension 1: Te taha wairua (spiritual dimension) Dimension 2: Te taha hinengaro (psychic dimension) Dimension 3: Te taha tinana (bodily dimension) Dimension 4: Te taha whānau (family dimension)	N/A

*For models and frameworks with more than one manuscript, data is presented here from the more recent manuscript. Where manuscripts were published in the same year, authors were contacted to determine manuscript for data extraction.

†Author provided additional information.

§This visualization was found in subsequent publications after the original manuscript describing the model/framework was published.

‡The name, though absent from the reviewed manuscript, was found in subsequent publications.

**Table 5:** Stated purpose and directions for future research.

Article citation	Model name	Purpose	Future directions
Camacho et al. (74)	N/A	Facilitate inágofli’e’ and alofa for Queer and Transgender Pacific Islander (QTPI) to combat settler colonialism’s impacts on well-being Promote anti-colonial recovery by centering Indigenous ways of being Restore QTPI connectedness to culture, families, and communities Contribute to sociocultural and social justice implications for decolonial futures inclusive of QTPI	Focus on violence as a health outcome and violence prevention for QTPI communities Explore PI individuals living in their Indigenous homelands and other diasporic spaces to improve generalizability Examine the experiences of other QTPI communities beyond CHamoru and Sāmoan Encourage other QTPI communities to build upon the model developed in this study
Johnson et al. (143)	The Ngaruroro model of Māori wellbeing	Formalize a model of Māori wellbeing grounded in the lived experiences of Māori people Identify sources of wellbeing for Māori individuals Serve as a foundation for developing a self-report Māori wellbeing measure Describe wellbeing in terms of interconnected themes (here tāngata, te taiao, taonga tuku iho, tinana, wairua, ngākau, matea, mana)	Thoroughly examine Māori cultural sources such as waiata, haka, mōteatea, pūrākau, whakataukī, and pepeha to provide context and insights for wellbeing research Collectively theorize through wānanga, interviews with tohunga, and analyses of historical documents Investigate ancestral knowledge related to waiora
Shea (76)^†^	The Living Well Model	Current models aren’t appropriate for community	Create a short form and a child/adolescent version of a measurement tool Take different components of the model and create educational/community programming to help promote living well (e.g., online repository of recipes that use traditional myaamia foods)
Paul et al. (77)	The Piikani Well-being Index (PWI)	Provide a holistic approach to understanding well-being, encompassing human health, agriculture, cultural systems, and more Guide community research agendas and action Define health and well-being from a local perspective Aid tribal decision-makers in resource allocation and planning Support Indigenous governance and sovereignty	Address data gaps within the Blackfeet Nation by identifying variables for future surveys Conduct more advanced statistical analyses to explore correlations at a sub-Nation scale Implement longitudinal studies and strategic planning through integrated community-led health, agriculture, and land use surveys Monitor and update the Piikani Well-being Index (PWI) with community engagement
Fletcher et al. 50	The IQI model of health	Identify and describe community and culturally relevant concepts and processes that lead to health and well-being Describe Inuit cultural concepts of health and well-being in relation to health determinants and community living Better understand how conditions and resources in communities contribute to the health of people Identify sources of strength and resilience in each community in response to health challenges Measure and describe community health and well-being across all 14 communities in Nunavik Provide data and information to develop community action plans and interventions to respond to the health and well-being needs of Nunavik Convey culturally familiar sentiments, meanings, and embodied conditions that constitute health Shift understanding of health from a Western perspective to one grounded in Inuit epistemology and lived experience	Further refine and apply of the IQI model in complex health systems Explore how social interaction and comfort are integrated into public health interventions Community-led research initiatives using multifaceted listening methodologies
Smith et al. (67)	The Good Spirit, Good Life tool and framework	Develop a culturally informed quality of life (QoL) tool for older Aboriginal Australians Understand the Aboriginal Australian worldview of QoL Identify factors that enhance QoL in older Aboriginal Australians Develop questions to encompass QoL priorities Assess face validity of the tool Optimize QoL by focusing on 12 key factors that enhance the inner spirit Inform policy makers, service providers, and families about Aboriginal perspectives on QoL	Implement quantitative validity testing of the draft tool Develop the tool into a preference-based measure for health and aged care economic evaluation Assess the reliability and construct validity of the draft tool and a version for family members/carers Develop a framework, strategies, and a training guide
Kanaʻiaupuni et al. (70)	The Pua Model: A Native Hawaiian perspective on well-being	Present a conceptual model of the dimensions of well-being from a Native Hawaiian perspective with the Pua Model Provide a holistic framework to assess the well-being of adults, families, communities, young children, and school-age children. Strengthen the foundation of Hawaiian culture-based education to rebalance systems and address inequities Provide high-quality learning experiences that meet community needs and promote well-being	Investigate factors contributing to the diminishing share of Native Hawaiians in public schools and strategies to address this trend Research effective educational interventions to improve academic achievement and postsecondary education rates among Native Hawaiian learners Examine the need for quality early learning programs and affordability, including funding resources and support systems Investigate strategies to reduce economic disadvantage among Native Hawaiian families
Dion et al. (53)	N/A	Explore Inuit conceptualizations of wellness Document wellness practices carried within communities Explore mechanisms by which practices influence wellness Document Inuit approaches and their underlying principles and practices in relation to self-determination	Strengthen Inuit representation and governance in health systems leadership Align systems of care with Inuit knowledge and lifeways, from development, to implementation, and evaluation Continue community-driven documentation of Inuit knowledge and practices
Panapa et al. (55)	The Ola Lei Framework	Contribute to understanding wellbeing in a Pacific context by specifying enabling activities Guide policy and strategic planning at organizational and national levels Link government planning with local values and practices Facilitate collaboration between formal and informal health sectors	Integrate traditional healing practices with biomedical healthcare to enhance collaboration and resources for wellbeing Develop a complementary relationship between traditional healers and biomedical practitioners Use the Ola Lei Framework to guide policy development and strategic planning
Cardinal and Pepler (52)	The Community Journey of Change model	Map Indigenous communities’ journeys from violence to wellness through relational determinants of health Represent actions for communities to move towards wellness Inform policies, programs, and services to restore health and wellness Guide organizations in supporting Indigenous communities’ journeys to wellness Address historical and ongoing harms of colonization Provide guidance for culturally relevant pathways to restore relational determinants of health	Indigenize the Canadian Red Cross’ violence prevention programming using the model Pilot and evaluate the model across Canada Continue walking alongside and learning with Indigenous communities in mobilizing for change and restoring relational determinants of health Promote systems change through Indigenous leadership and the creation of ethical space
Garvey et al. (64)	The Fabric of Aboriginal and Torres Strait Islander Wellbeing model	Present a new conceptual model of wellbeing for Aboriginal and Torres Strait Islander adults Describe the interrelationship of wellbeing dimensions Inform the development of a holistic wellbeing measure for health services and policy makers Prioritize Aboriginal and Torres Strait Islander voices and perspectives Enhance understanding of wellbeing from an Aboriginal and Torres Strait Islander perspective	Develop and validate a holistic measure of wellbeing based on the Fabric of Aboriginal and Torres Strait Islander Wellbeing conceptual model Further engage with the community to present the model back to participants
Odom et al. (73)	Pilinahā: The Four Connections Framework	Contribute to the body of work on community and Indigenous well-being Foster a more meaningful and effective health system by reshaping it to accommodate diverse experiences and healing pathways Encourage others to develop their own community paradigms for health and well-being Honor and uplift Indigenous insights and voices in the health system Build a toolkit for applying Pilinahā Create a health system that values all providers and places, integrating traditional healing practices	Refine Pilinahā to address spirituality and its translation into intentional practices in varied settings Develop metrics that focus on growing connections rather than just healthcare services Engage in community processes to clarify perspectives and frameworks for health that may resemble or differ from Pilinahā Continue to build a toolkit of questions, practices, and metrics for applying Pilinahā through collaborative storytelling and research
Kyoon-Achan et al. (48)	First Nations Mental Wellness Framework	Promote understanding of wellbeing in the context of sociocultural realities of First Nation communities Achieve mental wellbeing by and for First Nation people as part of primary healthcare Challenge paternalistic views by emphasizing community responsibility and active participation Advocate for collaboration and community involvement in healthcare solutions Help First Nation communities move from colonization towards balance and wellbeing Articulate cultural practices for mental wellbeing	Implement and evaluate the framework in community-based wellness interventions Utilize the framework to guide the alignment of funding and policy decisions with community priorities Apply the framework to evaluate mental health services, ensuring cultural alignment Continue research and framework development by and for First Nations peoples – both the greater collective, as well as individual communities
Kūkulu Kumuhana Planning Committee (69)	Kūkulu Kumuhana	Improve Native Hawaiian wellbeing through meaningful discussions among community leaders Address wellbeing at individual/family, community, and system levels Focus on cultural identity, spirituality, and collective wealth through themes like ‘Āina Momona, Waiwai, ‘Ōiwi, Ke Akua Mana Recapture and apply traditional Native Hawaiian values Develop a culturally relevant wellbeing framework for a healthy and thriving community	Fill data gaps regarding Native Hawaiian wellbeing from a Hawaiian worldview Develop a shared framework for wellbeing and educate the public on wellbeing’s key elements Explore dimensions like ‘Āina Momona, Waiwai, and ‘Ōiwi Integrate traditional values into modern practices Foster ongoing dialogue and collaboration among stakeholders
Wong-Wilson (71)	Wong-Wilson’s New Native Hawaiian Well-Being Model	Build on previous models of Native Hawaiian well-being • Reflect the symbiotic relationship of Native Hawaiians to the ‘āina and spirituality • Illustrate Hawaiian identity as complex, multi-faceted, multi-dimensional, multi-generational, and holistic • Center Native Hawaiian well-being in relation to the physical, psychological and spiritual world	• Research long-term success for Native Hawaiians at Hawai‘i’s Community Colleges • Benefit Hawaiian researchers through publication of Pono Hawai‘i research methodology and the Native Hawaiian Well-Being Model • Support and mentor other Hawaiian scholars in achieving their higher education goals
Cairney et al. (66) Cairney and Abbott (80)*	The Interplay Wellbeing Framework	• Understand the interplay between key influences on wellbeing to drive effective policy and service delivery • Map, investigate, and assess interactions between culture, community, empowerment, education, employment, health, and wellbeing • Use for engagement, planning, evaluation, communication, decision-making, and partnerships • Address deficiencies in evidence to inform policy decisions • Monitor and map progress in education, employment, health, and wellbeing • Identify areas for investment to improve wellbeing	• Expand the current research to offer cumulative value for wellbeing in remote Aboriginal and Torres Strait Islander communities • Explore potential expansion as a long-term, longitudinal, and nationally representative study • Present the research as a preliminary study with potential for future phases
Ontario’s Aboriginal Health Access Centres (51)	AHAC Model of Wholistic Health and Wellbeing	• Represent common value systems that frame the work of Ontario’s Aboriginal Health Access Centres toward healthy communities	• Utilize the value systems represented in the model to frame the work of the AHACs toward healthy communities
Ministry of Health (49)	First Nations Mental Wellness Continuum Model	• Improve mental wellness outcomes for First Nations individuals, families, and communities • Create a comprehensive continuum of quality programs and services by adapting and optimizing existing mental wellness programs • Realign current programs and funding to make them more responsive and flexible to the needs of First Nations peoples • Shift from fragmented programming to a comprehensive mental wellness system based on an evidence-based continuum of care • Enhance collaboration and build partnerships among healthcare providers and jurisdictional partners to meet the needs of First Nations people	• Continuously build a comprehensive evidence base for mental wellness programs and services in different communities • Conduct research founded on Indigenous knowledge and culture to bridge gaps and inform policy development • Ensure First Nations control over data collection processes • Develop an evaluation plan to support ongoing improvement of implementation processes • Explore culturally relevant indicators to center Indigenous perspectives in mental wellness
Gee et al. (65)	Social and Emotional Wellbeing from an Aboriginal and Torres Strait Islanders’ Perspective	• Define social and emotional wellbeing (SEWB) from an Aboriginal and Torres Strait Islander perspective • Clarify the relationship between SEWB, mental health, and mental health disorders • Emphasize the importance of social, cultural, historical, and political determinants in shaping SEWB • Provide a framework for understanding and applying SEWB principles and domains at a local level • Outline the nine guiding principles of the 2004 SEWB framework	• Clarify the conceptualization of SEWB and mental health to better understand their differences and intersections • Investigate how to promote resilience and strengths within Aboriginal and Torres Strait Islander communities • Further develop the SEWB framework to ensure its effective implementation and impact on health policy
Te Moananui-Makirere et al. (61)	The Te Ara Whakapikiōranga Framework	• Develop and sustain wellbeing • Guide the reclamation of practice wisdom within whānau • Support practice with whānau to develop and sustain wellbeing • Inform and develop whānau-centred practices	• Generate further thinking about the development and evidencing of whānau-centred practices and frameworks for the future
Kingsley et al. (63)	Exploratory Framework for Aboriginal Victorian peoples’ wellbeing	• Understand the relationship between Victorian Aboriginal peoples and their traditional land (Country) and its impact on wellbeing • Articulate positive and negative factors affecting Aboriginal health • Provide a holistic understanding of wellbeing, encompassing physical, social, emotional, political, cultural, and environmental aspects • Move beyond conventional wellbeing models • Explore the connection between Country and wellbeing for future applications and debates • Tackle future environmental and health issues by promoting connection to the natural environment	• Serve as a starting point for future application and debate of the developed framework • Address issues like climate change and urbanization • Support collaborative efforts among academics, policymakers, and communities • Advocate for natural environment connection using the framework • Investigate increased interaction with nature for better protection and health outcomes
Drabek (75)	Qik’rtarmiut Sugpiat’stun Sug’ucirpet: The Kodiak Alutiiq People’s Way of Being Human	• Promote culturally-relevant education practices. • Show how the Alutiiq traditional worldview is still a part of a continues knowledge stream • Offer a holistic perspective on the unity of the various elements, or spheres of life, and consider how aspects overlap or exist within the others	• Co-develop curricula based upon the study’s findings • Revitalize Alutiiq oral traditions, and reintegrate such traditions into formal education systems • Develop an anthology of Kodiak Alutiiq stories, accessible to teachers and students • Support collaborative work between community and educators
New South Wales Government (62) New South Wales Government (79)*	The Wellbeing Framework	• Develop understanding of current community wellbeing • Set goals and actions to improve community wellbeing • Facilitate negotiation with government and partners • Empower communities to identify and build upon strengths • Monitor and review effectiveness	• Promote ongoing community-government collaboration for wellbeing planning and monitoring, with the framework and toolkit as a foundation • Pilot and refine toolkit through community feedback
Mark and Lyons (59)	Te Whetu	• Explore Māori spiritual healers’ views on healing and healing practices in Aotearoa/New Zealand • Explore implications for understandings of MBS, health, illness and wellbeing • Create an expanded holistic model of Māori health and wellbeing	• Investigate beliefs of lay Māori, a more diverse population of healers, and other Māori health practitioners. • Explore the process of becoming a Māori healer. • Explore Māori concepts of MBS, spirituality, and communicating with ancestors/spiritual sources • Investigate topics like healing processes of Māori rongoā, romiromi, mirimiri, and incidences of family transgressions being passed down through the generations
Australian Bureau of Statistics (68)	The Indigenous Wellbeing Framework	• Provide a broad approach to understanding Indigenous wellbeing • Enhance Indigenous statistical information collection and analysis • Serve as a holistic guide for measuring wellbeing and reviewing priorities • Understand interactions within families and communities • Identify data gaps and provide a reporting structure	• Identify gaps in data for possible inclusion in future surveys • Develop new statistical measures based on the framework • Refine and expand the framework to more comprehensively reflect Indigenous wellbeing
Murray (60)	Te Punga Oranga	• Enhance health and wellness of kaimahi and tauira • Cater to the needs of Indigenous people in Aotearoa • Assist kaimahi in transforming their lives and those of their whānau • Educate and improve overall wellness • Benefit whānau, iwi, hapū, and tauira • Display commitment to kaimahi wellbeing and success	• Embed holistic wellbeing as a lasting institutional and community value • Continue influencing cultural change toward Indigenous models of wellness
McGregor et al. (1, 78)	Ho‘oulu Lāhui Aloha ‘Raising a Beloved Nation’ Ecological Model of Native Hawaiian Wellbeing	• Identify priorities for intervention services and program and various levels of the Native Hawaiian nation • Recognize the wealth of existing cultural and ecological resources • Recognize the strengths of traditional practices that perpetuate the Hawaiian culture • Identify how and where research can best be utilized	• Guide research efforts that will strengthen Native Hawaiian ‘ohana, community and nation well-being • Create research methodologies that accurately assess Native Hawaiian needs and encourage the development of a body of Native Hawaiian-centered research • Address paradigms of knowledge production, dissemination, and legitimacy
Durie (58)	Te Pae Māhutonga	• Promote health among Māori people by involving elders and accepting alternate goals and methods • Inform and improve health services and education by incorporating Māori values • Increase ethnic awareness and sensitivity in health services • Improve health services by incorporating Māori attitudes and experts into Western health systems	• Involve Māori elders in widescale interventions to promote health among Māori people • Explore implications of Māori health concepts for the development of health services and education in New Zealand • Address limitations in contemporary health services, particularly in the spiritual dimension, by incorporating Māori attitudes and experts into Western health systems
van Uchelen et al. (54)	N/A	• Provide an alternative approach to aboriginal mental health planning focusing on Indigenous knowledge and strengths • Highlight how First Nations people conceptualize wellness and emphasize existing strengths • Support and build on Indigenous resources to promote well-being in a culturally appropriate manner • Serve as a model for others to promote aboriginal well-being	• Utilize findings to support and build upon existing community strengths • Consult with community on an ongoing basis to ensure supportive efforts are aligned with current needs and priorities
Rezentes (72)	The Lōkahi Triangle	• Depict the concept of lōkahi, meaning achieving unity through harmony with self and others, nature, and God(s) • Emphasize interdependence between self and others, connection to ‘āina, relationship to God(s) and the spiritual world • Achieve holistic health by practicing interconnectedness and interdependence through the never-ending process of balancing the self with others and the physical and spiritual worlds	• Understand and incorporate Hawaiian cultural values such as lōkahi, aloha, and ‘ohana in psychological and healing work
Ministry of Health Ontario (49)	An Aboriginal Framework for Wholistic Health and Well-Being	• Provide broad direction and guidelines for Aboriginal involvement in health planning and service delivery • Improve the health of Aboriginal individuals, families, communities, and nations through equitable access to healthcare and culturally appropriate services • Support self-determination in health with financial and human resources for community-designed programs • Address health through a conceptual framework of life cycle, wholistic health, and continuum of care	• Research is required to support planning and informed decision-making regarding First Nation/Aboriginal communities’ health • Specific research is required to expand and enrich the knowledge and understanding of First Nation/Aboriginal communities’ health, particularly wholistic and traditional approaches to health and well-being, health determinants and conditions and the etiology of disease within the First Nation/Aboriginal population
Pere (57)	Te Wheke	• Share ancient teachings of Hawaiki for general publication • Educate about universal teachings without boundaries • Promote understanding and respect for all cultures and religions • Preserve and transmit cultural heritage • Make teachings accessible to a wider audience	• Engage with ancestral knowledge through lived experience - “feel the magic”
Durie (20)	Te Whare Tapa Whā^‡^	• Enhance wellbeing by increasing Māori participation in society • Promote autonomy • Create a climate for realizing human potential	• Integrate Māori values and the wisdom of elders into healthcare planning and delivery • Embed more integrative and culturally grounded health interventions into Aotearoa’s health systems, especially those incorporating a spiritual dimension

*For models and frameworks with more than one manuscript, data is presented here from the more recent manuscript. Where manuscripts were published in the same year, authors were contacted to determine manuscript for data extraction.

†Author provided additional information.

‡While this name was not provided in the manuscript reviewed, it was located in later publications.

**Table 6:** Original definitions of wellbeing or equivalent term.

Article citation	Model name	Definition of wellbeing
Johnson et al. (143)	The Ngaruroro model of Māori wellbeing	“The active process of being well in relation with (1) here tāngata, (2) te taiao, and (3) taonga tuku iho, making lifestyle choices that are conducive to the health of your (4) tinana and (5) wairua while cultivating a balanced ngākau (7), fulfilling matea, and (8) exercising your mana.”
Kanaʻiaupuni et al. (70)	The Pua Model: A Native Hawaiian perspective on well-being	“For Native Hawaiians, well-being manifests when we find pono (balance) and lōkahi (harmony) among the many aspects of our lives.”
Dion et al. (53)	N/A	Inuit wellness is defined by underlying principles that inform solutions to the social, family, and individual issues found in Inuit culture and knowledge. This is interwoven with, “ways of being together and of taking care of each other that are profoundly relational and inscribed within an intergenerational perspective focused towards the future.”
Panapa et al. (55)	The Ola Lei Framework	“The Tuvaluan view of health intertwines relational, economic, physical and spiritual dimensions of life, offering both explanatory utility and practical guidelines for living well.”
Wong-Wilson (71)	Wong-Wilson’s New Native Hawaiian Well-Being Model	“Native Hawaiian Well Being, therefore, is defined as a person’s sense of pono and kuleana within their ‘ohana, kaiaulu, lāhui, and ‘āina and in relation to their ‘aumakua and akua. Based on Kuleana (responsibilities); Pono (harmony); ʻOhana (family); Kaiaulu (Community, extended family); Lāhui (nation); ʻĀina (Land and ocean); Nā ʻaumakua (Ancestors) and akua (Gods)”
Ministry of Health (49)	First Nations Mental Wellness Framework	Mental wellness: “Balance and interconnectedness is enriched as individuals have: PURPOSE in their daily lives whether it is through education, employment, care-giving activities, or cultural ways of doing; HOPE for their future and those of their families that is grounded in a sense of identity, unique Indigenous values, and having a belief in spirit; a sense of BELONGING and connectedness within their families, to community, and to culture; and finally a sense of MEANING and an understanding of how their lives and those of their families and communities are part of creation and a rich history.”
Durie (58)	Te Pae Māhutonga	Oranga (wellbeing): “Not only about a secure cultural identity, or an intact environment, or even about the avoidance of risks. It is also about the goods and services which people can count on, and the voice they have in deciding the way in which those goods and services are made available.”
Rezentes (72)	The Lōkahi Triangle	Holistic health: The “never-ending process of balancing and harmonizing oneself with others and with the physical and spiritual worlds. Thus, by extension, lōkahi is holistic health.”
Durie (20)	Te Whare Tapa Whā^‡^	Health: “A Māori perspective sees health as a four-sided concept, representing four basic tenets of life. There is a spiritual component, a psychic component, a bodily component and a family component. On the marae these are referred to as ‘te taha wairua’, ‘te taha hinengaro’, ‘te taha tinana’ and ‘re taha whanau’. Together these components blend to form an integrated and comprehensive model for health.”

^‡^While this name was not provided in the manuscript reviewed, it was located in later publications.

### Search results

#### Description of models or frameworks

Table 1 presents a description of the characteristics of the 33 included models and frameworks.

Publication outlets for the selected manuscripts covered a wide range of academic disciplines, including interdisciplinary/transdisciplinary (see Table 1).

**Figure 2:**
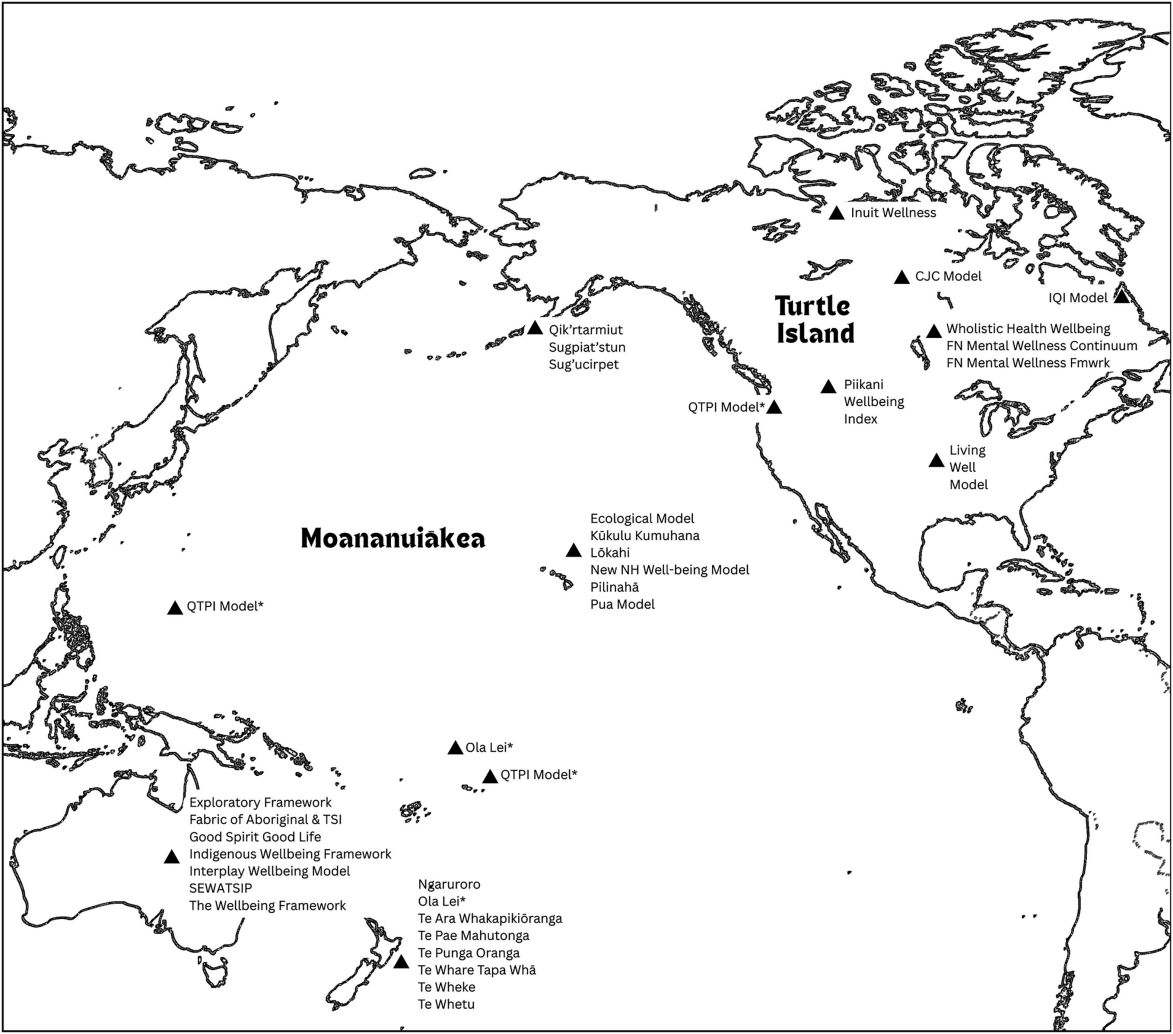
Map of models and frameworks from Turtle Island and Moananuiākea.

The 33 models included in this review originated from Indigenous communities in Turtle Island (*n* = 12) and Moananuiākea (*n* = 21). Places in which the frameworks were developed included Canada (*n* = 8) (19, 48–54), Aotearoa (*n* = 8) (20, 55–61), Australia (*n* = 7) (62–68), Hawaiʻi (*n* = 6) (1, 69–73), the United States (*n* = 3) (74–76), Tuvalu (*n* = 1) (55) (see Figure 2).

Indigenous communities were described as Aboriginal Peoples (*n* = 9) (49, 51, 62–68), Māori (*n* = 7) (20, 56–61), Kānaka Maoli or Kānaka ʻŌiwi (i.e., Native Hawaiians; (*n* = 6) (1, 69–73), First Nations Peoples (*n* = 6) (19, 48, 49, 51, 52, 54), Torres Strait Islander Peoples (*n* = 4) (64–66, 68), and Inuit Peoples (*n* = 3) (50, 52, 53). Other communities included Myaamiaki (i.e., Miami Nation; *n* = 1) (76), Amskapi Piikani (i.e., Blackfeet Nation; *n* = 1) (77), Sugpiaq (i.e., Alutiiq- Alaska Native; *n* = 1) (63), and Tuvaluan Peoples (*n* = 1) (55), and QTPI (i.e., Queer and Transgender Pacific Islanders based in Washington) (*n* = 1) (74).

Many of the models used qualitative methods (*n* = 17) (48, 50, 52–56, 59, 61, 63, 64, 66, 67, 71, 73, 74, 77). Other manuscripts drew upon lived experience, community consultations, or document review (*n* = 16) (1, 20, 49, 51, 57, 58, 60, 62, 65, 68–70, 72, 75, 76), Indigenous research methods (i.e., Indigenous Research, Indigenist Collaborative Research, Kaupapa Māori Methodology, Whānau-centred Research; *n* = 6) (56, 61, 64, 71, 73, 74), Participatory Action Research (PAR; *n* = 3) (53, 66, 67), Community-Based Participatory Research (CBPR; *n* = 3) (48, 50, 66), quantitative methods (*n* = 2) (66, 77), and mixed methods (*n* = 3) (66, 71, 77). See Table 1 for study design by source.

Participant information varied across models; 18 articles reported sample sizes ranging from under 20 to 7,000+, and participant roles included community members, Elders, spiritual healers, and decision-making bodies (see Table 1).

Tables 2, 3 describe 28 commonly identified aspects of Indigenous wellbeing and their frequency. The two most frequently referenced aspects were relationality and culture. All models and frameworks (*n* = 33) included relationality and culture. The two least frequently included aspects of Indigenous wellbeing were time as cyclical (*n* = 8) (1, 49, 51, 52, 57, 70, 73, 74) and outcomes of wellbeing (*n* = 11) (1, 20, 51, 52, 54, 56, 58, 60, 61, 64, 66).

#### Imagery used to portray model or framework

Details about imagery are summarized in Table 4. Articles employed both culturally grounded metaphorical imagery (*n* = 19) and diagrams (*n* = 11), with circular or radial designs most common (*n* = 14), reflecting the holistic, interconnected nature of Indigenous wellbeing.

#### Intended model or framework purposes

Table 5 provides a detailed description of the purposes and future directions of the models. Across settings and peoples, the model purposes cluster around four shared aims: (1) re-centering Indigenous determinants of health; (2) restoring self-determination and community control; (3) building practice and system capacity; and (4) strengths-based transformation over deficit remediation.

##### Re-centering Indigenous determinants of health

Most models were explicitly designed to uplift cultural identity, spirituality, land/‘āina/country, kinship, and history as core determinants of wellbeing (63, 65, 69, 70, 72). Their purposes position wellbeing as relational and ecological rather than narrowly biomedical. Many models specifically aim to challenge existing health and wellbeing frameworks (*n* = 7) (48, 50, 63, 64, 74–76) and privilege Indigenous voices. For example, the IQI Model of Health seeks to shift from Western understandings of health to ones grounded in Inuit epistemology and experience (50). Others aims include: re-center Indigenous determinants of health through exploration, promotion, and preservation of Indigenous perspectives (*n* = 11) (50, 54, 57, 59, 65, 67–72), broadly aiming to define or understand wellbeing from Indigenous perspectives (67, 70) and cater to the needs of Indigenous communities (60).

##### Restoring self-determination and community control

Purposes of the frameworks emphasize sovereignty, governance, and local agenda-setting (*n* = 9) (20, 48, 49, 51, 55, 62, 66, 74, 77). These frameworks call for decolonial healing and solidarity as important aspects of wellbeing, and seek to restore self-determination and autonomy for Indigenous communities. One example is the Piikani Well-being Index (PWI) (77), which seeks to guide research community agendas and action, aid tribal-decision makers in resource allocation, and support Indigenous governance and sovereignty. Further, purposes also highlight linking government planning with local values (55), empowering communities (62), and promoting autonomy (20).

##### Building practice and system capacity

A large subset of models are purpose-built to guide service planning and delivery, workforce, and partnerships (*n* = 11) (1, 49, 51, 52, 55, 58, 62, 64, 66, 68, 73). Some models specifically target the development of measurements and tools for these purposes (*n* = 6) (50, 64, 66–68, 77). Such work was intended to ground decisions, action, and tools in Indigenous understandings of wellbeing. For example, The First Nations Wellness Continuum was developed to create quality programs and services by optimizing existing wellness programs in Ontario (51). For measurement/tools, the Ngaruroro Model of Māori Wellbeing (56) provided the groundwork for a self-report measure of Māori Wellbeing.

##### Strength-based transformation over deficit remediation

Purposes consistently highlight assets and position the models as means to grow them (*n* = 8) (1, 50, 54, 56, 60, 62, 66, 70). This approach contrasts with biomedical orientations that emphasize deficit remediation. Instead, models seek to identify and build upon existing sources of thriving, such as traditional practices and knowledge, cultural and ecological resources, and community resilience. For example, Fletcher (50) identifies sources of strength and resilience in Inuit communities. Similarly, one purpose of Ho‘oulu Lāhui Aloha (78) was to highlight the wealth of existing resources and the strengths of Hawaiian traditional practices.

#### Differences across purposes

Across identified purposes for the models, there were four main categories of difference that were important in the wider discussion of intended uses. These areas of difference were: (1) level of action targeted, (2) primary function of the model, (3) population focus, and (4) sector anchor.

##### Level of action targeted

There were four main levels of action targeted across the models: individual (*n* = 4) (58, 60, 67, 74), family (*n* = 5) (49, 51, 60, 61, 69), community (*n* = 11) (1, 50, 52, 54, 56, 60, 62, 66, 70, 77, 79), system and policy (*n* = 4) (48, 51, 66, 68). Notably, many models present purposes that include multiple levels (1, 49–51, 69, 70).

##### Primary function of the model

Primary model functions aligned with four domains: conceptual orientation (*n* = 9) (53, 54, 57, 59, 63–65, 71, 72), planning and governance (*n* = 8) (48, 49, 51, 52, 55, 62, 73, 77), measurement, indices, and tools (*n* = 8) (51, 56, 64, 67, 68, 73, 76, 77), and issue-specific transformation (*n* = 3) (63, 70, 71).

##### Population focus

Many models focused on broader Indigenous adult and community populations (*n* = 20, First Nation communities, Māori people, Native Hawaiians) (1, 20, 48, 49, 51–59, 68, 69, 71–73, 76, 77). Others focused on specific groups, such as: older Aboriginal adults (67); urban First Nations peoples (54), remote Aboriginal and Torres Strait Islander communities (50, 66), Queer and Transgender Pacific Islanders (QTPI) (74), Māori whānau (61), and Native Hawaiian students and education personnel (70, 71).

##### Sector focus

Specific sectors were sometimes the focus of the frameworks, including health (*n* = 5) (49, 51, 55, 65, 73), education (*n* = 3) (70, 71, 75), economy, policy, and statistics (*n* = 11) (51, 52, 55, 56, 62, 64–66, 69, 75, 77), and an interplay of multiple sectors (*n* = 6) (55, 63, 64, 67, 73, 77).

#### Future directions

Future directions were identified in 33 manuscripts and organized into six overarching themes: (1) implementation and systems integration (*n* = 11) (49–52, 55, 58, 65, 71, 73, 76, 77), (2) development of Indigenous-led measures and indicators (*n* = 8) (51, 56, 64, 67, 68, 73, 76, 77), (3) education and workforce development (*n* = 9) (51–53, 58, 69–71, 75, 76), (4) strengthening Indigenous governance and data sovereignty (*n* = 14) (1, 19, 48, 50, 52–54, 56, 64, 69, 71, 73, 77), (5) intergenerational knowledge transmission (*n* = 7) (20, 55–59, 75), and (6) cross-sector collaboration and policy transformation (*n* = 7) (19, 55, 63, 65, 69, 75, 77). See Table 5 for future directions categories.

##### Implementation and systems integration

Future directions often emphasized implementing frameworks of wellbeing across systems and settings, and exploring their potential to inform policy (*n* = 11) (e.g., 73, 76, 77). These models suggest Indigenous wellbeing frameworks should be translated into service delivery systems, ensuring they are implemented under Indigenous governance and community control. Models are positioned to guide strategic planning and program development (55, 76, 77), policy development (19, 55, 65), and implementation within health, education, and other systems (50, 55, 58, 71, 73).

##### Development of Indigenous-led measures and indicators

Many models highlight the need for culturally grounded, valid, and context-specific metrics that capture holistic wellbeing (e.g., spiritual, relational, ecological) and replace Westernized indicators (*n* = 8) (e.g., 19, 56, 64). In response to this need, future directions often included the development and validation of new holistic wellbeing measurement tools (56, 64, 67, 68, 76), the implementation of new integrated surveys into future research studies (77), and the development of new metrics and indicators for wellbeing that center Indigenous perspectives (19, 73).

##### Education and workforce development

Future directions emphasize embedding Indigenous frameworks into public and global health curricula to support education and workforce development (*n* = 9) (e.g., 51–53). Models aim to build Indigenous leadership pipelines across sectors (52, 53), inform educational programming (58, 69, 75, 76), and support the long-term success of Indigenous scholars (70, 71).

##### Strengthening Indigenous governance and data sovereignty

Many future directions focused on shifting existing paradigms toward Indigenous governance (*n* = 15) (e.g., 52, 64, 69). Models call for Indigenous authority in decision-making (52, 53), Indigenous community participation and leadership in research processes (*n* = 13) (e.g., 48, 50, 52), data stewardship (1, 19, 48, 71, 77), and the co-governance of programs (58, 62).

##### Intergenerational knowledge transmission

Frameworks often stress continuity across generations in their descriptions of future directions (*n* = 7) (e.g., 55, 59, 75). This includes engaging with and upholding ancestral knowledge (56, 57, 59), traditional practices (55) and oral tradition (75). Future directions also point toward the integration of wisdom from Elders (20, 58) and intergenerational healing processes (59).

##### Cross-sector collaboration and policy transformation

Next steps for many of the models entail adaptation for multi-sectoral action and policy transformation (*n* = 8) (e.g., 19, 63, 77). Other models encourage collaboration between Indigenous communities, policymakers, academics and educators, and providers (55, 63, 69, 75), and seek to link health to the environment (77) in order to improve community wellbeing. Future directions also point to policy development (19, 55, 65).

#### Definitions of Indigenous wellbeing

Only nine of the 33 articles provided an original definition of wellbeing for their respective communities (20, 50, 52, 54, 55, 57, 59, 69–71), using varied terminology including wellness, hauora, and oranga (see Table 6).

### Synthesis of findings

Across the models included, there were notable similarities in the conceptualizations of wellbeing. While these models captured insight from different nations, cultures, and disciplines, synthesis of the data yielded seven main themes: (1) holistic integration of all life domains; (2) spirituality and the ongoing presence of ancestors; (3) culture and preservation of tradition; (4) collective wellbeing and relationality; (5) reciprocal relationship with land as a source of identity, health, and responsibility; (6) use of place-specific Indigenous metaphors and language; and (7) empowerment and self-determination. These themes were drawn from the identified aspects of wellbeing (Table 2), the outlined constructs and organizing categories of the models (Table 4), and the purposes and future directions (Table 5).

#### Holistic integration of all life domains

For Indigenous communities, wellbeing encompasses several distinct but interconnected domains (Table 4). These domains include physical and mental health (*n* = 24) (e.g., 19, 20, 62), emotional (*n* = 8) (e.g., 48, 51, 52), spiritual (*n* = 19) (e.g., 1, 75, 76), cultural (*n* = 16) (e.g., 69, 74, 77), economic and governmental (*n* = 15) (e.g., 50, 55, 60), collective wellbeing (*n* = 26) (e.g., 67, 68, 73) and environmental (*n* = 15) (e.g., 59, 63, 71). Other domains include ancestral connection (*n* = 10) (e.g., 56, 63, 69), identity (*n* = 10) (e.g., 54, 57, 74), philosophy and ethics (*n* = 2) (73, 75), power or autonomy (*n* = 7) (e.g., 62, 66, 72), food or food sovereignty (*n* = 3) (50, 55, 77). See Table 4 for full coverage by domain. For Indigenous communities in this study, wellbeing is not only multidimensional, but dependent upon harmony and balance between dimensions.

The theme of holistic integration of all life domains is evident in aspects of Indigenous wellbeing (Table 2), organizing categories for the models (Table 4), and purposes and future directions (Table 5) across all included models (*n* = 33). For aspects of Indigenous wellbeing, holism (*n* = 31) (e.g., 52, 60, 61), interconnectedness (*n* = 30) (e.g., 50, 53, 70) were among the most frequently captured. Ecological aspects (*n* = 19) (e.g., 1, 51, 67) were also reported in over half the models.

The imagery used to illustrate the frameworks reflected holistic wellbeing, for example, through culturally specific metaphors for wellbeing such as woven fabric or constellations to represent interconnected or multidimensional structures (48, 51, 56, 58, 64) multiple overlapping parts (e.g., Venn diagrams; see Table 4) (53). Beyond visual representation, models were often explicitly organized into multiple categories, described as: dimensions (1, 20, 50, 57, 70, 72), domains (66, 68), themes (54, 56), tenets (76), indicator areas (77), necessities and factors (67), principles (53), central qualities (55), foundations (52), aspects (59, 64), connections (65, 73), levels (69), spheres (71, 75), elements (51), wāhanga (61), characteristics (62), rau (60), and key tasks (58) that formed each framework.

Holism is also evident in the frameworks’ stated purposes and future directions, with articles advocating for holistic understanding of wellbeing which guide multilevel and multisystem collaboration (19, 49, 56, 63–66, 68–73, 75, 77, 80).

#### Spirituality and ongoing presence of ancestors

Spirituality is described as present in all aspects of Indigenous wellbeing, underscoring the deeply rooted connection between self, the natural world, and the spiritual realm (1, 19, 48–52, 54–56, 58–61, 63, 65–67, 69–74, 76). Related to spirituality is connection to ancestors; many frameworks emphasize the continuity of ancestors and ancestral knowledge as key to wellbeing (*n* = 11) (1, 56, 57, 59, 61, 63, 65, 69, 71, 73, 75). This connection to ancestors is specifically manifested in responsibilities to ancestors (71) and the breath of life from ancestors (57). These facets are evident in aspects of wellbeing depicted (see Table 2), organizing categories (see Table 4), and purposes and future directions (see Table 5) of many frameworks.

Spirituality is among the most commonly identified aspects of Indigenous wellbeing (*n* = 28) (1, 19, 20, 48–52, 54–61, 63, 65–67, 69–76). The majority of frameworks also emphasized intergenerational aspects of wellbeing (i.e., including ancestral wisdom and continuity; *n* = 27 (1, 19, 20, 48–51, 53–62, 65, 67, 69–76).

In examining the constructs outlined in the frameworks, spirituality, and continuity of ancestors emerge as critical in organizing categories, many frameworks categorizing spirituality as a key dimension contributing to wellbeing, and connection to ancestors as having an important influence on wellbeing (20, 48, 49, 51, 52, 54–57, 59, 60, 65, 67, 69, 70, 72, 75, 76, 78).

The purposes and future directions of the frameworks also highlight spirituality and continuity of ancestors (20, 49, 57–59, 67, 69, 71, 72, 75). Specifically, some models aim to center wellbeing in relation to the spiritual world (71), optimize quality of life by enhancing the spirit (67), explore spiritual healers’ views on wellbeing (59), and engage with ancestral knowledge through lived experiences (57).

#### Culture and preservation of tradition

Culture and preservation of tradition emerged as a key theme among several models. Connection to culture and access to traditional practices is critical to Indigenous understandings of wellbeing (*n* = 21), (1, 19, 48, 51, 53–56, 58, 62–70, 74, 75, 77). All models included in this review (1, 19, 20, 48–77) emphasized culture as an aspect of wellbeing. Another commonly reported aspect of wellbeing in the models was cultural sovereignty (*n* = 22) (1, 19, 20, 50–53, 56, 58–62, 64–71, 74).

Culture and tradition are common organizing categories with cultural identity and connection to culture frequently highlighted as key domains of wellbeing (1, 51, 56, 62, 65–70, 77) (see Table 4). Alternatively, culture is conceptualized as a facet of life (64, 74) or both a domain and a determinant of wellbeing (48, 56, 63, 73, 75).

The purposes and future directions of the models also highlight culture and preservation of tradition as a theme, specifically the importance of understanding wellbeing from a cultural perspective, integrating traditional healing practices into systems of care, and perpetuating connection to Indigenous culture (see Table 5) (1, 49, 55, 63, 65, 69, 74, 75).

#### Collective wellbeing and relationality

Across the included frameworks, collective wellbeing and relationality emerge as key themes in Indigenous wellbeing. Wellbeing is not only understood at the individual level, but also at the family, community, and nation level (*n* = 23) (1, 19, 20, 48–50, 53, 54, 56, 57, 59–62, 64–66, 68, 69, 71, 73, 74, 76). The self does not exist in isolation; connection with others is fundamental to wellbeing, and relationality also extends to the elements of the natural world (1, 19, 20, 48, 52, 53, 56–61, 63, 66, 69, 71, 73, 77) and ancestors (1, 56, 59, 61, 63, 65, 69, 73, 75). Collective wellbeing and relationality are evident at multiple levels of the frameworks, including identified aspects of wellbeing (Table 2), organizing categories and construct descriptions (Table 4), and purposes and future directions (Table 5).

All models and frameworks included relational aspects of wellbeing (*n* = 33). Most emphasized community (*n* = 30) (e.g., 56,61,62) and collectivist dimensions (*n* = 29) (e.g., 55,60,77). Family was also a frequently captured aspect of wellbeing (*n* = 25) (e.g., 52,53,72). Further, land as family was commonly identified as an aspect of wellbeing (*n* = 19) (e.g., 58,59,71); reflecting a unique way of being wherein Indigenous humans are inclined toward relationality, not just with each other, but also with the natural environment.

The organizing categories and construct descriptions within the frameworks highlight collective wellbeing and relationality as central to Indigenous wellbeing, with community and family connection often conceptualized as central components and/or sources of wellbeing (see Table 4). Frameworks also describe relationality, family, and community connection as aspects of life (64, 74), core cultural values (77), connections (73), and outcomes (51).

Relationality and collective wellbeing are also embedded in the purposes and future directions of the models. Many of these aims point toward uplifting views of wellbeing through a collective lens and understanding and addressing wellbeing on a community and family level (1, 20, 48–50, 53, 54, 60–64, 66, 68, 69, 72, 73, 76, 77).

#### Reciprocal relationship with land as a source of identity, health, and responsibility

Connection to land, environmental stewardship, and place-based identity arose as a common theme across Indigenous wellbeing models. Land and place hold deep significance to many Indigenous communities, and wellbeing is understood as sustained by a strong connection to and responsibility toward the land. As such, land stewardship (*n* = 22) (1, 19, 20, 48, 50–52, 56, 58–63, 65, 66, 69, 70, 73, 74, 76, 77) and land as family (*n* = 19) (1, 19, 20, 48, 52, 53, 56–63, 66, 69, 71, 73, 77) emerged as commonly identified aspects of wellbeing across the models (see Table 2). Models also often included ecological aspects of wellbeing (*n* = 19) (1, 19, 20, 50–52, 58–67, 69, 74, 76), indicating the importance of connection between individuals and the environment for wellbeing.

The theme of reciprocal relationship with land as a source of identity, health, and responsibility is evident in the organizing categories of the models, with land and environment emerging as common dimensions of wellbeing (1, 59, 60, 69, 71, 72) and connection with the environment as a determinant or theme of wellbeing (48, 53, 56, 70, 73, 75). Models highlight caring for the land as wellbeing indicators (77), knowledge competencies (76), values (75), and key tasks of health promotion (58). Relatedly, the concepts of place and country help shape Indigenous identity, a critical aspect of wellbeing (1, 56, 63, 73, 76).

Across model purposes and future directions, the act of describing wellbeing in terms of interconnected themes is a common purpose, and many models with this purpose include connection to the land and environment as key themes (56, 63, 69, 72, 77). Most models integrate land in some form, by highlighting the wealth of ecological resources (1), implementing agriculture and land use surveys into health data (77), or calling for advocacy for natural environment connection and the mitigation of environmental health issues (63).

#### Use of place-specific Indigenous metaphors and language

Across the reviewed frameworks, a key theme was the use of culturally-specific metaphors and language to structure and explain wellbeing concepts. Ancestral languages, stories, and teachings were alluded to or included directly in frameworks (*n* = 19) (1, 20, 50, 55–61, 69–77), highlighting the importance of revitalising and reconnecting with Indigenous languages to support wellbeing. Many of the included frameworks *(n* = 15) were named in an Indigenous language (20, 55–57, 59, 61, 69, 70, 72, 73, 75, 77). These titles were not always direct translations of wellbeing nomenclature; titles were often culturally significant concepts and metaphors, such as Lōkahi (harmony/balance) (72) and Te Pae Māhutonga (Southern Cross constellation) (58).

Some frameworks used Indigenous language to name their organizing categories (i.e., Wāhanga, Rau) (60, 61), while others used Indigenous language and metaphors for wellbeing related constructs, dimensions, themes, or determinants (1, 20, 50, 55–61, 69–77). For example, Fletcher et al. 50 in the IQI Model of Health describe three dimensions of wellbeing in Inuktitut (an Inuit language): Ilusirsusiarniq (bodily health), Qanuinngisiarniq (sense of well-being), and Inuuqatigiitsianiq (ideal form of social well-being). Models often featured cultural values in Native languages. For example, different models across Hawai‘i frequently described Hawaiian values (e.g., pilina, pono, lōkahi) as key to wellbeing (1, 69–73).

#### Empowerment and self-determination

Across Indigenous communities, empowerment and self-determination are considered fundamental to wellbeing, particularly to combat the detrimental and contemporary impacts of colonialism (*n* = 14) (e.g., 56, 63, 74). Many frameworks emphasize the desired capacity for self-governance, access to culture and country, and control over resources (*n* = 11) (e.g., 1, 65, 77). Sovereignty is critical to the understanding and pursuit of wellbeing, and different expressions of sovereignty comprise multiple commonly identified aspects of Indigenous wellbeing (see Table 2), including cultural sovereignty (*n* = 22) (e.g., 53, 60, 65), personal sovereignty (*n* = 20) (e.g., 49, 69, 74), political sovereignty (*n* = 17) (e.g., 56, 76, 77), and economic sovereignty (*n* = 12) (e.g., 58, 68, 70). See Table 2 for full coverage.

The theme of empowerment and self-determination is frequently captured through the outlined constructs and organizing categories of the frameworks, including principles related to self-determination, empowerment, and community resilience (see Table 4). Principles related to self-determination (e.g., institutional capacity, leadership and autonomy, access to cultural resources, connection to country, and wellbeing of the nation) are conceptualized as pathways to and domains of wellbeing (1, 53, 58, 62, 65, 68, 69, 77). Organizing categories of the models and frameworks also emphasize empowerment and community resilience (66, 67).

Purposes and future directions prioritize Indigenous governance and community empowerment (see Table 5). All models reflect the theme of community empowerment and self-determination by seeking to define and conceptualize wellbeing from an Indigenous perspective (e.g., 64, 65, 72), guiding systems-level action and agendas (e.g., 19, 51, 62), or serving as the foundation for Indigenous-centered measures and indices (e.g., 56, 64, 67). Specifically, many model purposes and future directions prioritize research autonomy and data stewardship for Indigenous communities (e.g., 50, 53, 77) and call for Indigenous authority in decision-making, programs, and services (52, 53, 58, 62).

## Discussion

This review identified 33 models from Turtle Island and Moananuiākea and confirmed seven common themes (see Results). Findings demonstrate that Indigenous wellbeing models function as coherent decolonial theories that challenge Western paradigms through distinct ontological, epistemological, axiological, and practical commitments. By employing an analysis from etic and emic views, we demonstrate how Indigenous wellbeing models can be compared across cultures while preserving their irreducible culture-specific meanings. This review also contributes to decolonial theory-building in public and global health by centering Indigenous knowledge systems as foundational theories in their own right.

No theories were included in the review, a finding that itself warrants interpretation. This is not a search artefact: ‘theory’ was an explicit search term, and theory language does appear in the literature (e.g., the KANU Theory of wellbeing was encountered but excluded as behavioral health-specific) (81). Notably, ‘model’ and ‘framework’ are equally Western constructs, suggesting authors were already navigating Western terminology selectively. Western scientific theory requires falsifiability, operationalizability, generalizability, endeavors that may not reflect the priorities of communities engaged in the work of articulating and uplifting Indigenous knowledge systems. That several future directions across included models explicitly prioritize measurement development suggests theory formalization may be the field’s next horizon.

### Situating findings in existing scholarship

This review builds upon recent syntheses of Indigenous health frameworks while making novel contributions. Previous reviews have identified fundamental differences between Indigenous relational-holistic conceptions and Western individualistic approaches (82), emphasized culture as central to Indigenous health care models (83), and called attention to gaps in culturally appropriate assessment tools (84, 85). Scholarship on Indigenous mental health has emphasized moving beyond cultural competence to address structural determinants and power imbalances (86), proposed centering Indigenous therapeutic traditions rather than adapting Western interventions (87), and developed frameworks for reimagining distress, wellbeing, treatment, and evaluation through Indigenous lenses (87). While this work has advanced the field, most reviews have focused on single countries or specific health domains, leaving a need for comprehensive cross-regional synthesis.

We make three primary contributions. First, we provide the first comprehensive cross-regional synthesis of Indigenous wellbeing models from Turtle Island and Moananuiākea, revealing both shared principles and essential diversity. Second, our etic/emic analytical framework navigates the tension between enabling comparison and avoiding epistemic violence by explicitly distinguishing comparable patterns from meanings that resist translation. Third, we organize models’ contributions to decolonial public and global health theory through ontology, axiology, epistemology, and praxis, demonstrating how Indigenous frameworks operationalize calls for structural transformation (88, 89) through concrete theoretical commitments. Unlike previous work treating these as culturally adapted Western models, we demonstrate they may be used as coherent public health theories.

### Historical evolution: from recognition to transformation

In analyzing how models have changed over the last 40 years, our review identified that early works sought to establish holistic health as foundational or to bring to light traditional wellbeing frameworks as alternatives to Western ones (20, 54). Over the last 30 years, models have become increasingly population-specific, taking an intersectional approach (67, 74), or increasingly sophisticated, offering innovative applications such as the integration of land stewardship, food sovereignty, and health measurement (50, 53, 77), or in some cases explicitly accounting for determinants such as colonization and violence (52, 63, 74). An exception to this trajectory over time is illustrated by the 2003 Ecological Model of Native Hawaiian Wellbeing which accounts for uniquely rich details in each of the named levels for that time (see Table 4). This evolution has critical implications for how public and global health engage with Indigenous knowledge. Early models generally established Indigenous perspectives as legitimate alternatives to Western frameworks; contemporary models demand that health systems fundamentally reorganize around Indigenous ontologies, epistemologies, and governance structures. This shift signals that Indigenous communities are no longer prioritizing inclusion in existing systems but asserting their authority to define health paradigms and transform health systems to center Indigenous governance and lifeways. Models developed in the past decade demonstrate that Indigenous communities are ready to lead this transformation—the question is whether institutions are prepared to follow.

In terms of broad categories of wellbeing elements (i.e., supports, detriments, states, and outcomes), most models exclusively illustrate supports and states of wellbeing in their visual models, while addressing detrimental determinants as part of the purpose or discussion sections (50, 55, 56, 62, 64, 66, 70, 73, 90). Many models illustrate domains in which both supports and detriments could factor (19, 48, 60, 65, 68, 72, 91), yet only four models visually illustrate detrimental determinants to wellbeing (52, 63, 67, 74). Where detriments were visualized, physical, mental, emotional, and spiritual domains were all represented, including explicit naming of colonization, violence, racism, the introduction of Christianity, loss of traditions, specific policies and treaties, destruction of land, policing of QTPI identity, and erasure (74). Several models note in their supporting text that colonialism and decolonization provides the context for processes of wellbeing (53, 61), the necessity of critical consciousness (understanding historical trauma and colonialism) as a principle or support of wellbeing (71, 77), acknowledge trauma or impacts of colonization in a secondary organizing category not displayed in the visual (56, 57, 59), or include the broad category of overcoming hardship or barriers as a main feature of the model (54). The variation in how models address colonial harms reflects different strategic choices: some emphasize Indigenous strengths to counter deficit narratives, while others insist that colonial violence must be named and visualized to guide structural intervention. Public and global health must learn to hold both approaches, recognizing that healing and liberation require both cultural revitalization and direct confrontation of ongoing colonial oppression.

While the seven resulting themes elaborated similarities across Indigenous experiences and worldviews, findings also underscore diversity in cultural expressions. Frameworks reviewed are uniquely articulated: Māori models center whānau, wairua, and manaakitanga; Native Hawaiian frameworks emphasize aloha, pono, and ‘āina; Aboriginal Australian models highlight country, cultural continuity, and empowerment; Turtle Island frameworks foreground kinship, the elements, and four directions. These differences reinforce that conceptions of wellbeing cannot be fully standardized - their rootedness in land and language is vital. Furthermore, even within each respective model’s community, there exists vast diversity (65). Some models reviewed are population-specific within an Indigenous community such as older Aboriginal Australians (67), Native Hawaiian community college students (71), urban-dwelling Aboriginal peoples (54), or remote Aboriginal and Torres Strait Islander communities (66). Such specificity underscores their contextually informed design, which in turn supports more reliable analysis of change in service delivery over time.

### Etic and emic perspectives on Indigenous models of wellbeing

We employ the anthropological distinction between etic (external/comparative) and emic (internal/culture-specific) perspectives to analyze these models at two levels. From an etic perspective, this synthesis reveals recurring constructs that align with established public and global health categories, including multidimensional wellbeing, social and collective health, environmental determinants, culture, spirituality, identity, and autonomy. These concepts enable comparison across diverse models and facilitate synthesis by translating heterogeneous Indigenous frameworks into a shared analytic vocabulary. For example, collective wellbeing and relationality resonate with social determinants and community-level health approaches, while land and environment are often categorized as ecological or contextual determinants of health. While this etic framing is analytically useful, these categories are necessarily approximate. Terms such as “spirituality,” “culture,” “environment,” and “empowerment” function as conceptual bridges rather than precise translations, and risk obscuring the distinct ontological and epistemological nuances embedded within Indigenous models.

At an emic level, Indigenous wellbeing models articulate a fundamentally deeper understanding of wellbeing that reshapes these etic categories. Rather than treating wellbeing as the sum of discrete domains, the models emphasize harmony, balance, and relationality as constitutive of health. Wellbeing is not located within the individual body or psyche, but emerges from dynamic relationships among people, families, communities, land, ancestors, and spiritual worlds. Visual metaphors such as woven fabric, constellations, waterways, medicine wheels, and nested circles are not merely illustrative; they encode Indigenous ontologies in which interdependence and reciprocity are foundational (48, 51, 56, 58, 64).

The theme of holistic integration demonstrates this distinction clearly. Emically, these frameworks emphasize harmony and balance among domains, rejecting fragmentation of health into discrete components. Unlike Western models emphasizing either individual hedonic (satisfaction, positive emotions) or eudaimonic (purpose, meaning) wellbeing, Indigenous models emphasize the interconnectedness of all beings’ health and flourishing: human and more-than-human, land, ancestors past, and generations into the future (Table 2 and Table 4). While some models include hedonic aspects such as happy living (Ii-ta-mii-pa-ta-pi-yoip) (77), happiness (iaia) (55), and humor (75, 76), these are inextricably intertwined with the wellbeing of family and land, emphasizing balance and connection within the ecology of organizing elements rather than narrow individual focus (20).

Where purpose and meaning are emphasized (19, 48, 64, 73), interconnectedness and balance are enriched (19), dignity and respect through one’s work is supported (48, 64), and kuleana (responsibility, privilege) to one’s ancestors past and generations forward is uplifted (73, 92). When one behaves with respect for others, land, and self (57, 63, 64, 67, 75, 77), and strives to do good work (55, 76, 77) life is lived well, benefitting the entire ecology. This relational ontology has profound implications for decolonial health promotion as it resists the absorption of Indigenous holism into Western biomedical or biopsychosocial models and instead advances wellbeing as emerging from the dynamic alignment of life domains.

Spirituality, ancestral continuity, land, and culture further illustrate this emic/etic distinction. Etically, these can be categorized as common wellbeing domains; emically, however, they are not separable aspects but ever-present dimensions permeating all facets of life. Ancestors are active ongoing presences that confer knowledge, responsibility, and vitality (see Table 4). Land is not an environmental context external to the self but, for many cultures, a relation of kinship that shapes identity, health, and moral obligation (see Table 4). Culture is not an external influence on health but a living system of knowledge, practice, and governance. Indigenous languages, values, and metaphors function as epistemic foundations rather than communicative embellishments, asserting Indigenous authority over the meaning and measurement of wellbeing.

Frameworks use metaphors, language, and symbols from their respective lands and honored cultural symbols such as the octopus (55, 57), plants (50, 60, 63, 70), a crescent moon (73), a star (59), rohe potae (traditional tribal lands) (91), a clay pot (74), a protective talisman (75), and fabric from weaving traditions (64), illustrating ontological embeddedness. Self-determination and empowerment are emically understood as collective, structural, and intergenerational processes tied to sovereignty, governance, control over resources, and authority in decision-making, including research and data stewardship.

The contrast between etic approximations and emic meanings has important implications for decolonial public and global health. While etic frameworks enable synthesis and comparison, Indigenous wellbeing models challenge dominant assumptions about wellbeing, calling for relational, place-based, and pluriversal approaches. Used judiciously, etic tools can make emic knowledge systems visible without collapsing them into categories, underscoring the need for structural transformation to advance health equity.

### Contributions to decolonial theory

Given that self-determination is a vital element in most models reviewed, Indigenous wellbeing is at once a local public health imperative *and* a part of the global health ecosystem. We therefore draw from *both* public health and global health discourse on decolonialism here. The compelling call for decolonial theory in these fields reflects a critical movement aimed at transforming how health knowledge is constructed and utilized in efforts to support marginalized communities. In its early stages of development, decolonial theory in public and global health focused on acknowledging Eurocentric paradigms and their harms while present trends indicate greater theory building and application.

#### Decolonial ontology

Coloniality is not simply historical but an ongoing structure that produces hierarchies of being and health (93, 94). Indigenous models of wellbeing echo calls from decolonial public and global health (6, 95–97) to explicitly acknowledge colonialism as a determinant of health and advance Indigenous ways of being that transcend dominant categories of disease and risk (48, 52, 53, 59, 63, 74). Addressing these inequities requires more than redistributing resources; it demands an ontological shift in defining wellbeing as relational, ecological, and spiritual, concepts long marginalized by biomedicine. The Indigenous wellbeing models analyzed in the present review affirm and clarify these views in decolonial public and global health theory.

From an Indigenous ontology, health is constituted not only through individual physical states but through reciprocal relationships among body, mind, spirit, family, community, land, ancestors, and more-than-human relations. As illustrated in Table 2, the intergenerational continuity at the heart of Indigenous wellbeing models illustrates an ontology where notions of time are less linear and more circular or cyclical, reflecting Indigenous temporalities in which the past stands in front of us (98). This view also reflects a responsibility to make wise decisions in the present for the sake of future generations’ wellbeing. Indigenous models foreground more-than-human relations, further encoding social and cultural identity in enduring relationships with territories and ecosystems. Integrating human and environmental health emphasizes reciprocal ties between cultural vitality and ecological stewardship or kinship with land. Ontologically, these frameworks reject compartmentalized biomedical ontologies and embed in the foundations of health worldviews that support sustainability in an era of climate crisis (30).

Decolonial intersectionality (99) provides an important lens for the development of ontology in decolonialism, centering the views and voices of Majority World and Indigenous peoples, recognizing how colonialism imposed binary gender systems that disrupted relational understandings of identity and belonging (100–103). By revealing how such binaries sustain colonial hierarchies and environmental exploitation (104, 105), this framework advances a vision of sustainable global justice, highlighting how ecological balance, land sovereignty, and environmental justice are essential dimensions of collective wellbeing (14, 91, 106, 107). Two wellbeing models in particular recognize the intersectionality of health determinants by exploring wellbeing for older Aboriginal Australians (67) and QTPI living in diaspora (74). Through an intersectional lens, both frameworks highlight detriments to wellbeing for Indigenous peoples with identities more vulnerable in colonized contexts: a lack of safety and security (for family members and selves), and identity-based violence. These conceptions expand upon decolonial efforts in public and global health by underscoring the importance of an intersectional lens, one which requires inclusivity of the most vulnerable within Indigenous communities.

#### Decolonial axiology

Understanding Indigenous values and ethics, the central foci of axiology, is critical for the development of decolonial theory in public and global health. Example models elaborate on values and principles: Manitoba First Nations values and principles underlying approaches to mental wellbeing (48), Māori community values (55), Inuit intergenerational transmission of values to sustain connection with wellbeing of others (53), Aboriginal and Torres Strait Islander values that shape views of wellbeing (64), an ethical sphere and values named for each of the other spheres of wellbeing for the Alutiiq (75), and the Myaamiaki community value system to guide worldview and behaviors (76). Across these models, axiology is not an abstract philosophical layer but an operational guide for living well, in right relation, with land, kin, non-human beings, governance systems, and future generations. Decolonial theory in public and global health must orient itself to these axiological commitments as they provide guidance to lead the field from individualism to relational accountability, from pathology to cultural strength, and from externally imposed metrics to community-governed indicators. These frameworks foreground the importance of axiology in decolonial theory, illustrating how ethical systems shape ways of seeing, knowledge systems, and lifeways with the potential to inform the decolonizing of global and public health systems. They demonstrate that transforming health systems requires more than adding Indigenous content—it requires centering Indigenous ethics as the basis for decision-making, evidence, intervention design, and the redistribution of power.

#### Decolonial epistemology

These Indigenous models of wellbeing contribute to decolonial epistemologies by elevating Indigenous ways of knowing and knowledge production as legitimate and authoritative. Authors from global health highlight colonial genealogies and call for “epistemic disobedience” to delink knowledge production from Eurocentric dominance (94). This emphasis on epistemic justice has expanded into legal and ethical debates, with attention to how global health law and governance must be restructured to recognize plural knowledges and dismantle rigid hierarchies (93, 108). Centralizing the wisdom of marginalized groups, crediting and compensating Indigenous experts, ensuring authorship and leadership opportunities, and health training embeds Indigenous epistemologies as critical correctives to epistemic violence in public and global health (109, 110).

While re-defining wellbeing is first an ontological declaration of what exists as “well,” it is also an epistemological commitment to how that wellbeing is known, experienced, enacted, and measured, embedding the knowledge systems through which these ontologies are expressed. Indigenous models of wellbeing contribute to epistemological frames of decolonizing in global and public health by asserting that knowledge is generated through story, ceremony, chanting, and intergenerational transmission, with an emphasis on Indigenous language as the medium (Table 4). They uplift community-defined indicators of success, Indigenous-led research, and data sovereignty (19, 50, 61, 77). Even the Indigenous methods used to define these models provide examples for applied use of decolonial epistemology: the palm tree mapping exercise (74), whānau-centred methodology (61), Kaupapa Māori research approach (56), Elder knowledge sharing (53), and yarning groups (67). While these models suggest respect for diverse worldviews (57, 76), they reject Western universality in health and affirm Indigenous definitions as central for Indigenous peoples (49–51) and as a part of the multiplicity of epistemologies among humankind. Findings from these models suggest that decolonial epistemologies in public and global health do not simply de-link from Eurocentric knowledge dominance to embrace plural perspectives (94), they center Indigenous ways of knowing, and therefore defining, measuring, and evaluating.

#### Decolonial praxis - implications for practice and policy

In both global and public health, praxis is a central site where decolonial theory is translated into action. While a shift from rhetoric to practical reforms is underway in global health (e.g., restructuring funding architectures, diversifying authorship, embedding accountability mechanisms) (34, 94, 111), interventions should be carefully evaluated by their ability to avoid reproducing colonial power relations (112). Parallel efforts are apparent in public health including education-focused reforms promoting alliance-building, community mobilization, and authorship practices that recognize Indigenous expertise (95, 109). Indigenous models of wellbeing align with and expand upon these efforts, emphasizing how Indigenous knowledge and practice must be at the center of decolonizing efforts (31–34, 53). This imperative is anchored in what framework authors themselves envisioned: across all 33 included models, future directions converged on six action priorities that map directly onto decolonial praxis: implementation and systems integration, development of Indigenous-led measures and indicators, education and workforce development, strengthening Indigenous governance and data sovereignty, intergenerational knowledge transmission, and cross-sector collaboration and policy transformation (see Table 5). Along these lines, decolonial praxis in Indigenous wellbeing models is enacted through Indigenizing: prioritizing community-led governance and Indigenous leadership (the most broadly envisioned future direction), culturally grounded healing techniques, and land-based practices that embed relationality and spirituality in daily life (see Table 4). These practices additionally extend decolonial praxis by grounding health interventions in sovereignty, relational ethics, and place-based obligations rather than in externally imposed biomedical agendas (111, 113). Importantly for programming designed to serve both Indigenous and non-Indigenous communities, accepting everyone is a core value, suggesting an Indigenous approach to wellbeing is an inclusive one (75, 77). Together, these examples highlight how praxis in decolonial health is made increasingly concrete using Indigenous wellbeing models, Indigenizing at the levels of global and local implementation, measurement, workforce transformation, and governance.

Indigenous wellbeing models offer policy pathways for decolonizing climate action to support all living beings. While approaches like One Health link human, animal, and environmental wellbeing (114), they maintain Western epistemology and risk advancing technocratic agendas (115). Indigenous models instead ground sustainable climate action by framing health, environment, and governance as co-constituted domains of relationality, reciprocity, and collective responsibility (30). These frameworks shift climate governance from preserving capitalist order toward restoring pluralism, kinship between people, land, and multispecies ecologies, addressing root causes of health and ecological inequities (82, 116–118). Centering Indigenous governance systems can transform climate accountability structures, ensuring decisions reflect community-defined ethics of reciprocity and self-determination (119). When policy centers Indigenous wellbeing models, a decolonial turn in public and global health becomes possible, shifting from hierarchies of control toward connection, balance, ecological justice, and collective thriving for future generations.

Indigenous models of wellbeing have the potential to dismantle inequitable colonial structures, positioning cultural identity, language, and land relations as protective determinants that counter hierarchical dominance (see Table 2) (6). By framing wellbeing through spiritual, familial, and environmental interdependence rather than biomedical determinants, they reassert Indigenous lifeways and challenge the adequacy of mainstream approaches to capture Indigenous realities. Indigenous wellbeing frameworks thus function as tools of decolonial resistance, shifting ontologies, epistemologies, axiologies, and praxes to reshape dynamics of power and privilege in public and global health—a transformation mapped in the six future directions that Indigenous authors themselves articulated across four decades and diverse nations.

### Limitations

The present scoping review is limited by several factors which may inform a measured reading of this work, as well as implications for future directions. This review reports on wellbeing models, frameworks, and theories that are limited to individuals, families, and communities, a choice intended to support aims of relevance and feasibility for a single review. Given the abundance of models or frameworks focused on youth, a future review focused on these community members is indicated. Additionally, while the health of the land or an environmental level or dimension of wellbeing is a feature in all models reviewed here, we did not include models or frameworks which elaborated on this aspect of wellbeing as the *primary* focus. Throughout the review process, many articles that primarily focused on a geographical approach to wellbeing were excluded (120–139). Future research could explore models that are primarily focused on the environmental level. Further, we were only able to review manuscripts written in English as this was the research team’s primary language, potentially restricting findings. A final limitation relevant to search terms is the exclusion of the word “health” in our final search string as this produced a number of manuscripts not feasible for a single scoping review. Even after excluding this search term, several manuscripts described models focused on specific diagnoses such as chronic disease or mental and behavioral health challenges (81, 140). These were excluded to ensure both specificity and feasibility for this review; future reviews might focus on health frameworks and models.

While steps were taken to Indigenize the research process, this review was conducted within the limitations of a Western knowledge system, which communicates information through existing literature commonly derived through an empirical research context. The present scoping review was designed and initiated prior to publication of Indigenous-informed scoping review methodology guidance (141). Retrospective assessment indicates alignment with all criteria outlined in that framework, with the exception of a formal Advisory Council. The all-Indigenous research team, engagement in critical reflexivity, Indigenous research paradigm, and community-centered approach collectively reflect the spirit and intent of an Indigenous approach to scoping reviews. The included corpus reflects heterogeneity in evidence type, encompassing peer-reviewed articles alongside theses, government policy documents, community planning documents, and a blog post. Consistent with JBI methodology, no formal quality appraisal was conducted; however, we argue this heterogeneity is epistemologically necessary: privileging peer-reviewed journals alone would reproduce the colonial knowledge hierarchies this review seeks to disrupt, excluding frameworks developed through Indigenous governance and community-led processes. Notably, Shea (76) represents a model whose community has not yet approved full academic release; the blog post reflects Indigenous data sovereignty in practice, and accuracy of representation was confirmed directly with the authors.

The evidence base in this review is predominantly qualitative and context specific. Some studies lacked detailed demographic or methodological information. While there is a strong convergence of principles across the included studies, the transferability of these findings to other contexts would require careful local adaptation and ongoing community engagement. Finally, we note that wellbeing in many of these cultures is viewed as a natural outcome from Indigenous lifeways. To honor this ontology, when authors described guidance for lifeways in model form as a means to ensure ecological wellbeing, we included them in this review. We also note the use of the terms “model,” “framework,” or “theory” as limiting the search findings to publications where authors chose to use these Western terms for illustrating ideas. While this was a necessary step to reduce the sheer number of manuscripts ultimately analyzed, this also meant we excluded articles which may have described a model or framework but did not name it as such.

### Future directions

The Indigenous wellbeing models analyzed in this review provide actionable frameworks ready for translation into public health practice, policy, and research. Future work should focus on implementation, measurement, workforce development, and governance across multiple domains.

#### Implementation and systems transformation

Research should pilot interventions based on these frameworks across diverse contexts, evaluating their effectiveness in strengthening relational, cultural, and ecological determinants of health. This includes adapting public health systems to support Indigenous governance and leadership, and embedding these frameworks into national and regional policies that align with principles of self-determination and relational wellbeing. Multisectoral collaboration will be essential to translate these models from theory into sustained health promotion and service delivery.

#### Measurement and data sovereignty

Creating culturally grounded measurement and data systems that capture the holistic dimensions of Indigenous health remains a critical priority. Several frameworks reviewed here emphasize the need for valid indicators reflecting spiritual, relational, and environmental aspects of wellbeing, moving beyond conventional biomedical or economic metrics. Building on emerging work in Indigenous measurement (13, 81, 142–147), future studies should continue refining indicators, advancing Indigenous data sovereignty initiatives, and integrating these measures into population health surveillance and evaluation systems. This will enable governments and public health agencies to monitor progress through Indigenous-defined outcomes, affirming that the health of people, land, and spirit are inseparable.

#### Workforce development

Transforming the public health workforce is essential for operationalizing Indigenous frameworks. Embedding these models into educational curricula can prepare practitioners, policymakers, and researchers to engage with Indigenous worldviews, ethics, and methodologies. Training initiatives should prioritize cultural safety, relational accountability, and place-based practice, while building pathways for Indigenous leadership in health research, program design, and evaluation. Such efforts will ensure continuity between community knowledge systems and institutional practice, creating conditions for systemic change rather than symbolic inclusion.

#### Governance and context-specific research

Sustained partnerships and governance structures that uphold Indigenous authority in decision-making are essential for embedding these models within broader public and global health systems. This requires long-term funding for community-led research, co-governance of health services, and policy mechanisms that empower Indigenous peoples to define and monitor their own wellbeing priorities. Future reviews could examine Indigenous wellbeing frameworks within specific contexts - such as behavioral health, pandemic response, youth development, or frameworks centered primarily on environmental wellbeing - to identify how these models operate across distinct domains and inform targeted interventions.

## Conclusion

This scoping review addressed two aims: to map Indigenous models and frameworks of wellbeing from Turtle Island and Moananuiākea, and to contribute to decolonial theory-building in public and global health. Across 33 models spanning four decades, we identified seven core themes and demonstrated how Indigenous frameworks articulate distinct ontological, epistemological, axiological, and practical commitments that cannot be reduced to Western categories.

A primary contribution in this review is demonstrating that Indigenous wellbeing models provide decolonial theory, foundational paradigms grounded in interconnected relationality, spirituality, ancestral continuity, and land-based lifeways. Through our etic/emic analytical framework, we reveal both what enables comparison across diverse models and what remains irreducibly culture-specific, challenging public and global health to honor commensurability and difference simultaneously.

We acknowledge tensions inherent in cross-cultural synthesis. While our findings demonstrate profound diversity across and within Indigenous communities, any comparative analysis risks essentializing “Indigenous knowledge” as monolithic. Our etic/emic framework navigates this by distinguishing what enables comparison from what remains irreducible, though we recognize all synthesis involves interpretive choices. Similarly, we caution that calls for institutions to Indigenize or decolonize must involve structural power shifts and Indigenous governance, not merely incorporating Indigenous content into unchanged colonial systems.

The stakes extend beyond documenting disparities to realizing Indigenous-defined visions of wellbeing. Indigenous frameworks foreground the interdependence of people, land, and spirit, offering testable paradigms that move beyond Western determinants to Indigenous relations of health. They demonstrate how revitalizing Indigenous ways of being, seeing, and doing not only restores cultural integrity but transforms health systems toward equity and sustainability. The knowledge is present, the frameworks are developed, and the communities are ready, what remains is a choice by the institutions and systems entrusted with public and global health. The question facing the field is clear: Will public and global health transform to align with Indigenous knowledge systems and lifeways, or continue marginalizing paradigms that offer sustainable pathways to healing and justice?

## Footnotes

1
10.17605/OSF.IO/S65KG
