# Supplementary material for: A systematic scoping review for decolonial public and global health: Indigenous frameworks and models of wellbeing from Turtle Island and Moananuiākea
Source: Front Public Health. 2026 Jul 16;14:1809539. doi: 10.3389/fpubh.2026.1809539 (PMC13422500; doi:10.3389/fpubh.2026.1809539)
Supplement: Supplementary file 1 [file Table_1.DOCX]

**
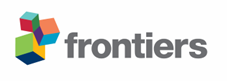
**

***Supplemental Material A. Positionalities of Authors***

Like the community members these models are designed for, **Joanne Qina**ʻ**au, PhD, MA, E-YT750** (she/they) is Indigenous (Kanaka ʻŌiwi, Ainu, Irish, Japanese, Portuguese) born and raised on Oʻahu in rural and urban single-parent homes. They are embodied spirit as daughter, aunty, friend, granddaughter, teacher, student, traveler, future ancestor. Their commitment to stewarding mauli ola and working toward equity on the path to collective liberation/ea began in 2006 and is informed by traumatic losses and violence in their ‘ohana due to settler colonial stress and huakaʻi of healing, learning, and teaching in Global Majority spaces in the Pacific, Asia, the Middle East, and Europe. As a clinical psychologist, Jo leverages transformative research as ritual, offering works such as this in an effort to heal backward for kūpuna and visioning forward to ho’omana Indigenized futures.

**Aubrey Yanger Mariano, MPH** is a CHamoru woman and scholar born and raised on Guåhan, and now nourished by the island of O‘ahu, Hawai‘i, the ‘āina of Kānaka ‘Ōiwi. Her research in Indigenous & Pacific decolonial and anti-carceral approaches to health is driven by lived experience navigating the health disparities impacting her CHamoru community, as a result of 500+ years of multi-empire colonization. As such, reclaiming health and wellbeing from an Indigenous lens is deeply personal to Aubrey, who seeks to center the strength and beauty of Indigenous peoples toward uplifting their collective wellness.

**Melissa K. Kahili-Heede, MLIS, MEd** is a Kanaka ʻŌiwi mother and haumana living on the island of Oʻahu in the US-occupied Kingdom of Hawaiʻi. Her views on mauliola are informed by a Kanaka ʻŌiwi sense of place and through the lens of aloha ʻāina and ʻike Hawaiʻi. She is still in the process of learning and unlearning, but proudly embraces the kuleana to work for and with Kānaka, always first turning to the source, ʻike kūpuna, and indigenous research methods for inspiration and guidance.

**Selah Kone** is Alaska Native (Sugpiaq/Unangax̂) and she is currently enrolled in the Indigenous and rural Health PhD program at Montana State University, which she is now completing remotely from her home community on Kodiak Island, Alaska. Her focus is on the intersection of Indigenous wellbeing and food sovereignty. Her goal in this work is to understand what it means to do research in an Indigenous way, by bringing her ancestors and the next seven generations along with her to explore what Indigenous wellbeing can look like from a community-determined perspective on wellbeing within a thriving food system.

**Caleb Rivera, MA** is a descendant whose ancestors practiced and sustained Indigenous wellbeing traditions for centuries, and as a community researcher, he brings both insider knowledge of Indigenous wellbeing concepts while understanding the reality that Indigenous people often face when their ways of knowing encounter systems built on colonial foundations. His position as both community member and community researcher creates opportunities for culturally grounded interpretation while also requiring ongoing reflection about the power dynamics inherent in academic knowledge production about Indigenous peoples. He approaches this work with kuleana to ensure that Indigenous wellbeing conceptualizations are presented in a pono way that serves rather than extracts from the communities whose ancestors have sustained these ways of knowing for generations.

**Cerila C. Rapadas,** **BA** is a Filipino and CHamoru woman born and raised on the island of Guåhan. She is humbled to conduct her clinical psychology doctoral training in Hawai‘i, āina of the Kānaka ‘Ōiwi, and to learn from Indigenous scholars, before returning to serve her home island and the broader Micronesian region. As a daughter of Guam, Cerila aims to incorporate Indigenous epistemologies, practices, and collectivist cultural frameworks into psychological treatments and measures. Through research, practice, and community engagement, she hopes to help improve the quality of treatment and research for serving Indigenous and local communities in the Pacific.

**Valerie J. Clack, MA, BCBA, LBA** (she/her) is an enrolled member of the Oglala Sioux Tribe raised in Northern California where she was taught intertribal traditions and protocols in the context of the Wintu and Pit River Nations. The Lakota and Oceti Ŝakowiŋ concept of Mitákuye Oyás'iŋ meaning, ‘we are all related’ or ‘all my relations,’ guides Valerie’s worldview as well as her participation in research benefitting indigenous and native communities. Just as she endeavors to be a good relative supported by ʻāina and Kānaka ʻŌiwi, Valerie strives to practice indigenous research methods in a good way. In her roles as a community member, researcher, behavior analyst, and parent, Valerie pursues wellbeing through the Lakota values of waóhola, wóksape, and wówauŋšila to envision a more equitable future.

**Shayla Chatto, PhD** positions herself as a Diné (Navajo) and N’dee (White Mountain Apache) scholar who understands wellbeing through the Diné teaching Hózhó, which is a good way of living and state of being, or wellbeing. Through her lens, she recognizes that Indigenous communities across North American and Oceania have distinct conceptualization and teachings of wellbeing/wellness that are grounded from peoples, places, oral stories, and more.

Kia ora tātou, ko **Finley Ngarangi Johnson** (he/him) tōku ingoa. I have both Māori (Rongomaiwahine, Ngāti Kahungunu, Rongowhakaata) and Indian (Yes, we ‘Mindians’ do exist) ancestral connections. The unique way in which I experience the world is shaped by generations of disconnection and a journey of cultural revitalisation. My undergraduate training in Māori studies, postgraduate training in critical cross-cultural research methods, and PhD thesis research in Indigenous Psychometrics has both nurtured my critical consciousness and provided me with tools to engage in indigenising praxis. This positionality of mine is marked by the privileges that I have been afforded simply by living as a cisgender, heterosexual, able bodied, neurotypical, lighter-skinned young male from a two-parent working class family in Aotearoa. Mauri ora!

**Michelle Aihina I^n^kish Holphokunna Johnson-Jennings, PhD, EdM,** is Choctaw nation tribal citizen and a distinguished American Indian clinical health psychologist with decades of expertise in harm and risk reduction and chronic disease prevention; including diabetes and cancer and addiction research, focusing on both substance use and food addiction. She currently serves as a Full Professor, Chambers-Hall Endowed Professor, clinical health psychologist and Co-Executive Director for the Indigenous Wellness Research Institute/IWRI, as well as Director for Indigenous Environmental Health and Land-based healing Division. Her work has been instrumental in advancing culturally responsive, community-driven health interventions that address the unique challenges faced by Indigenous populations across the globe. Grounded in her clinical training, Dr. Johnson-Jennings has dedicated much of her research to Indigenous women and children, particularly in the realm of increasing healthy diets and exercise through traditional land-based practices. Utilizing a community-based participatory research framework, she has employed quantitative, qualitative, and mixed-method approaches to explore and implement effective health strategies. Most importantly she is the mother to four amazing children.

**Mapuana C. K. Antonio,** is a Kanaka ʻŌiwi scholar whose positionality is inseparable from her aloha and kuleana to community and ʻĀina. Her lived experiences and commitment to health, healing, and wellbeing are grounded in the strengths, knowledge, and resilience of her communities. Trained in psychology, public health, community-based translational research, and Indigenous methodologies, she aims to (re)construct conceptions of health and wellbeing to better align with Native Hawaiian and Indigenous worldviews, ensuring our relations and ancestral knowledge pave the way for health and healing for the past, present, and future.
