# Supplementary material for: A systematic scoping review for decolonial public and global health: Indigenous frameworks and models of wellbeing from Turtle Island and Moananuiākea
Source: Front Public Health. 2026 Jul 16;14:1809539. doi: 10.3389/fpubh.2026.1809539 (PMC13422500; doi:10.3389/fpubh.2026.1809539)
Supplement: Supplementary file 2 [file Table_2.DOCX]

**
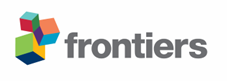
**

***Supplemental Material B. Search Strategy***

PubMed

7/17/23

| # | Keywords | # results |
| --- | --- | --- |
| 1 | (wellbeing[tiab] OR well-being[tiab] OR “quality of life”[tiab] OR wellness[tiab]) | 495,278 |
| 2 | ("indigenous framework"[tiab:~4] OR "Hawaiian framework"[tiab:~4] OR “Kanaka framework"[tiab:~4]OR "Maori framework"[tiab:~4] OR "Native framework"[tiab:~4] OR "Alaska Native framework"[tiab:~4] OR "Alaska Natives framework"[tiab:~4] OR "American Indian framework"[tiab:~4] OR "First Nation framework"[tiab:~4] OR "First People framework"[tiab:~4] OR "Aboriginal framework"[tiab:~4] OR "Inuit framework"[tiab:~4] OR "torres strait framework"[tiab:~4] OR "Chamorro framework"[tiab:~4] OR "Chamoru framework"[tiab:~4] OR "Micronesia framework"[tiab:~4] OR "Micronesian framework"[tiab:~4] OR "Palau framework"[tiab:~4] OR "Palauan framework"[tiab:~4] OR "Samoa framework"[tiab:~4] OR "Samoan framework"[tiab:~4] OR "indigenous model"[tiab:~4] OR "hawaiian model"[tiab:~4] OR “Kanaka model"[tiab:~4] OR "Maori model"[tiab:~4] OR "Native model"[tiab:~4] OR "American indian model"[tiab:~4] OR "Alaska Native model"[tiab:~4] OR "Alaska Natives model"[tiab:~4] OR "First Nation model"[tiab:~4] OR "First People model"[tiab:~4] OR "Aboriginal model"[tiab:~4] OR "Inuit model"[tiab:~4] OR "torres strait model"[tiab:~4] OR "chamorro model"[tiab:~4] OR "Chamoru model"[tiab:~4] OR "Micronesia model"[tiab:~4] OR "Micronesian model"[tiab:~4] OR "Palau model"[tiab:~4] OR "Palauan model"[tiab:~4] OR "Samoa model"[tiab:~4] OR "Samoan model"[tiab:~4] OR "indigenous theory"[tiab:~4] OR "hawaiian theory"[tiab:~4] OR “Kanaka theory"[tiab:~4] OR "maori theory"[tiab:~4] OR "Native theory"[tiab:~4] OR "Alaska Native theory"[tiab:~4] OR "Alaska Natives theory"[tiab:~4] OR "American indian theory"[tiab:~4] OR "First Nation theory"[tiab:~4] OR "First People theory"[tiab:~4] OR "Aboriginal theory"[tiab:~4] OR "Inuit theory"[tiab:~4] OR "torres strait theory"[tiab:~4] OR "chamorro theory"[tiab:~4] OR "Chamoru theory"[tiab:~4] OR "Micronesia theory"[tiab:~4] OR "Micronesian theory"[tiab:~4] OR "Palau theory"[tiab:~4] OR "Palauan theory"[tiab:~4] OR "Samoa theory"[tiab:~4] OR "Samoan theory"[tiab:~4] OR "indigenous knowledge"[tiab:~4] OR "hawaiian knowledge"[tiab:~4] OR “Kanaka knowledge"[tiab:~4] OR "maori knowledge"[tiab:~4] OR "Native knowledge"[tiab:~4] OR "Alaska Native knowledge"[tiab:~4] OR "Alaska Natives knowledge"[tiab:~4] OR "American indian knowledge"[tiab:~4] OR "First Nation knowledge"[tiab:~4] OR "First People knowledge"[tiab:~4] OR "Aboriginal knowledge"[tiab:~4] OR "Inuit knowledge"[tiab:~4] OR "torres strait knowledge"[tiab:~4] OR "chamorro knowledge"[tiab:~4] OR "Chamoru knowledge"[tiab:~4] OR "Micronesia knowledge"[tiab:~4] OR "Micronesian knowledge"[tiab:~4] OR "Palau knowledge"[tiab:~4] OR "Palauan knowledge"[tiab:~4] OR "Samoa knowledge"[tiab:~4] OR "Samoan knowledge"[tiab:~4] OR "Marshall islands framework"[tiab:~4] OR "marshallese framework"[tiab:~4] OR "Mariana islands framework"[tiab:~4] OR "Tahiti framework"[tiab:~4] OR "Tahitian framework"[tiab:~4] OR "Vanuatu framework"[tiab:~4] OR "ni-Vanuatu framework"[tiab:~4] OR "Melanesia framework"[tiab:~4] OR "Melanesian framework"[tiab:~4] OR "Polynesia framework"[tiab:~4] OR "Polynesian framework"[tiab:~4] OR "Oceania framework"[tiab:~4] OR "Pacific framework"[tiab:~4] OR "Pasifika framework"[tiab:~4] OR "Marshall islands model"[tiab:~4] OR "marshallese model"[tiab:~4] OR "Mariana islands model"[tiab:~4] OR "Tahiti fmodel"[tiab:~4] OR "Tahitian model"[tiab:~4] OR "Vanuatu model"[tiab:~4] OR "ni-Vanuatu model"[tiab:~4] OR "Melanesia model"[tiab:~4] OR "Melanesian model"[tiab:~4] OR "Polynesia model"[tiab:~4] OR "Polynesian model"[tiab:~4] OR "Oceania model"[tiab:~4] OR "Pacific model"[tiab:~4] OR "Pasifika model"[tiab:~4] OR "Marshall islands theory"[tiab:~4] OR "marshallese theory"[tiab:~4] OR "Mariana islands theory"[tiab:~4] OR "Tahiti theory"[tiab:~4] OR "Tahitian theory"[tiab:~4] OR "Vanuatu theory"[tiab:~4] OR "ni-Vanuatu theory"[tiab:~4] OR "Melanesia theory"[tiab:~4] OR "Melanesian theory"[tiab:~4] OR "Polynesia theory"[tiab:~4] OR "Polynesian theory"[tiab:~4] OR "Oceania theory"[tiab:~4] OR "Pacific theory"[tiab:~4] OR "Pasifika theory"[tiab:~4] OR "Marshall islands knowledge"[tiab:~4] OR "marshallese knowledge"[tiab:~4] OR "Mariana islands knowledge"[tiab:~4] OR "Tahiti knowledge"[tiab:~4] OR "Tahitian knowledge"[tiab:~4] OR "Vanuatu knowledge"[tiab:~4] OR "ni-Vanuatu knowledge"[tiab:~4] OR "Melanesia knowledge"[tiab:~4] OR "Melanesian knowledge"[tiab:~4] OR "Polynesia knowledge"[tiab:~4] OR "Polynesian knowledge"[tiab:~4] OR "Oceania knowledge"[tiab:~4] OR "Pacific knowledge"[tiab:~4] OR "Pasifika knowledge"[tiab:~4]) | 4,973 |
| 3 | #1 AND #2 | 330 |

Web of Science

7/17/23

<https://www.webofscience.com/wos/woscc/summary/5a0752b4-569c-438b-9c84-7a84702fe0a1-98673b72/relevance/1>

| # | Keywords | # results |
| --- | --- | --- |
| 1 | TS = (wellbeing OR well-being OR “quality of life” OR wellness) | 729,469 |
| 2 | TS = ((indigenous NEAR/4 (framework OR model OR theory OR knowledge)) OR ((Hawaiian* OR Kanaka) NEAR/4 (framework OR model OR theory OR knowledge)) OR (Maori* NEAR/4 (framework OR model OR theory OR knowledge)) OR (Native* NEAR/4 (framework OR model OR theory OR knowledge)) OR ("American Indian*” NEAR/5 (framework OR model OR theory OR knowledge)) OR ("Alaska Native*” NEAR/4 (framework OR model OR theory OR knowledge)) OR (“First Nation*” NEAR/4 (framework OR model OR theory OR knowledge)) OR ("First People*” NEAR/4 (framework OR model OR theory OR knowledge)) OR (Aborigin* NEAR/4 (framework OR model OR theory OR knowledge)) OR (Inuit* NEAR/4 (framework OR model OR theory OR knowledge)) OR ("torres strait” NEAR/4 (framework OR model OR theory OR knowledge)) OR (Chamorro* NEAR/4 (framework OR model OR theory OR knowledge)) OR (Chamoru* NEAR/4 (framework OR model OR theory OR knowledge)) OR (Micronesia NEAR/4 (framework OR model OR theory OR knowledge)) OR (Micronesian* NEAR/4 (framework OR model OR theory OR knowledge)) OR (Palau NEAR/4 (framework OR model OR theory OR knowledge)) OR (Palauan* NEAR/4 (framework OR model OR theory OR knowledge)) OR (Samoa NEAR/4 (framework OR model OR theory OR knowledge)) OR (Samoan* NEAR/4 (framework OR model OR theory OR knowledge)) OR ("Marshall islands" NEAR/4 (framework OR model OR theory OR knowledge)) OR (Marshallese NEAR/4 (framework OR model OR theory OR knowledge)) OR ("Mariana islands" NEAR/4 (framework OR model OR theory OR knowledge)) OR (Tahiti NEAR/4 (framework OR model OR theory OR knowledge)) OR (Tahitian* NEAR/4 (framework OR model OR theory OR knowledge)) OR (Vanuatu NEAR/4 (framework OR model OR theory OR knowledge)) OR (ni-Vanuatu NEAR/4 (framework OR model OR theory OR knowledge)) OR (Melanesia* NEAR/4 (framework OR model OR theory OR knowledge)) OR (Polynesia NEAR/4 (framework OR model OR theory OR knowledge)) OR (Polynesian* NEAR/4 (framework OR model OR theory OR knowledge)) OR (Oceania NEAR/4 (framework OR model OR theory OR knowledge)) OR (Pacific NEAR/4 (framework OR model OR theory OR knowledge)) OR (Pasifika NEAR/4 (framework OR model OR theory OR knowledge))) | 21,826 |
| 3 | #1 AND #2 | 820 |
| 4 | #1 AND #2 AND English (Languages) | 812 |
| 5 | #1 AND #2 AND English (Languages) and Article or Review Article or Early Access (Document Types) | 796 |

EBSCO - Academic Search Complete & CINAHL

| # | Keywords | # results |
| --- | --- | --- |
| 1 | (wellbeing OR well-being OR “quality of life” OR wellness)  AND  (indigenous OR hawaiian* OR kanaka OR maori* OR Native* OR “American indian*” OR “Alaska Native*” OR “Alaska Natives” OR “First Nation*” OR “First People*” OR Inuit* OR Aborigin* OR “torres strait” OR chamorro* OR Chamoru* OR Micronesia OR Micronesian* OR Palau OR Palauan* OR Samoa OR samoan* OR “Marshall islands” OR marshallese OR “Mariana islands” OR Tahiti OR tahitian* OR Vanuatu OR ni-Vanuatu OR Melanesia OR melanesian* OR Polynesia OR polynesian* OR Oceania OR Pacific OR Pasifika) N4 (framework OR model OR theory OR knowledge) | 902 |
| 2 | Limit to peer-reviewed | 853 |
| 3 | Limit to English | 841 |

ProQuest

07/17/2023

Limit to Peer-Reviewed, English

| # | Keywords | # results |
| --- | --- | --- |
| 1 | (wellbeing OR well-being OR “quality of life” OR wellness)  AND  (indigenous NEAR/4 (framework OR model OR theory OR knowledge)) OR ((Hawaiian* OR Kanaka) NEAR/4 (framework OR model OR theory OR knowledge)) OR (Maori* NEAR/4 (framework OR model OR theory OR knowledge)) OR (Native* NEAR/4 (framework OR model OR theory OR knowledge)) OR ("American Indian*” NEAR/5 (framework OR model OR theory OR knowledge)) OR ("Alaska Native*” NEAR/4 (framework OR model OR theory OR knowledge)) OR (“First Nation*” NEAR/4 (framework OR model OR theory OR knowledge)) OR ("First People*” NEAR/4 (framework OR model OR theory OR knowledge)) OR (Aborigin* NEAR/4 (framework OR model OR theory OR knowledge)) OR (Inuit* NEAR/4 (framework OR model OR theory OR knowledge)) OR ("torres strait” NEAR/4 (framework OR model OR theory OR knowledge)) OR (Chamorro* NEAR/4 (framework OR model OR theory OR knowledge)) OR (Chamoru* NEAR/4 (framework OR model OR theory OR knowledge)) OR (Micronesia NEAR/4 (framework OR model OR theory OR knowledge)) OR (Micronesian* NEAR/4 (framework OR model OR theory OR knowledge)) OR (Palau NEAR/4 (framework OR model OR theory OR knowledge)) OR (Palauan* NEAR/4 (framework OR model OR theory OR knowledge)) OR (Samoa NEAR/4 (framework OR model OR theory OR knowledge)) OR (Samoan* NEAR/4 (framework OR model OR theory OR knowledge)) OR ("Marshall islands" NEAR/4 (framework OR model OR theory OR knowledge)) OR (Marshallese NEAR/4 (framework OR model OR theory OR knowledge)) OR ("Mariana islands" NEAR/4 (framework OR model OR theory OR knowledge)) OR (Tahiti NEAR/4 (framework OR model OR theory OR knowledge)) OR (Tahitian* NEAR/4 (framework OR model OR theory OR knowledge)) OR (Vanuatu NEAR/4 (framework OR model OR theory OR knowledge)) OR (ni-Vanuatu NEAR/4 (framework OR model OR theory OR knowledge)) OR (Melanesia* NEAR/4 (framework OR model OR theory OR knowledge)) OR (Polynesia NEAR/4 (framework OR model OR theory OR knowledge)) OR (Polynesian* NEAR/4 (framework OR model OR theory OR knowledge)) OR (Oceania NEAR/4 (framework OR model OR theory OR knowledge)) OR (Pacific NEAR/4 (framework OR model OR theory OR knowledge)) OR (Pasifika NEAR/4 (framework OR model OR theory OR knowledge)) | 482 |

Google Scholar

6/11/2024

Limited to Articles

Split to meet 256 character limit

Two members of the research team (JQ and AM) reviewed the first eight pages of results (~80 records) using the same inclusion and exclusion criteria applied to database records; 42 records not already captured in the database search were advanced to full-text review.

| # | Keywords | # results |
| --- | --- | --- |
| 1 | (wellbeing\|well-being\|“quality of life”\|wellness) ("indigenous” AROUND (4) “framework"\|”model”\|”theory”\|”knowledge”)\|("Hawaiian" AROUND (4) “framework"\|”model”\|”theory”\|”knowledge”)\|(“Kanaka" AROUND (4) “framework"\|”model”\|”theory”\|”knowledge”) | **Total:** ~193k  **New on pp. 1-8:** 42 |
| 2 | (wellbeing\|well-being\|“quality of life”\|wellness) (“Maori" AROUND (4) “framework"\|”model”\|”theory”\|”knowledge”)\|(“Alaska Native" AROUND (4) “framework"\|”model”\|”theory”\|”knowledge”)\|(“Alaska Natives" AROUND (4) “framework"\|”model”\|”theory”\|”knowledge”) | **Total:** ~24k  **New on pp. 1-8:** 61 |
| 3 | (wellbeing\|well-being\|“quality of life”\|wellness) (“American Indian" AROUND (4) “framework"\|”model”\|”theory”\|”knowledge”)\|(“First Nation" AROUND (4) “framework"\|”model”\|”theory”\|”knowledge”) | **NONE** |
| 4 | (wellbeing\|well-being\|“quality of life”\|wellness) (“First People" AROUND (4) “framework"\|”model”\|”theory”\|”knowledge”)\|(“Aboriginal" AROUND (4) “framework"\|”model”\|”theory”\|”knowledge”)\|(“Inuit" AROUND (4) “framework"\|”model”\|”theory”\|”knowledge”) | **NONE** |
| 5 | (wellbeing\|well-being\|“quality of life”\|wellness) (“torres strait" AROUND (4) “framework"\|”model”\|”theory”\|”knowledge”)\|(“Chamorro" AROUND (4) “framework"\|”model”\|”theory”\|”knowledge”)\|(“Chamoru" AROUND (4) “framework"\|”model”\|”theory”\|”knowledge”) | **NONE** |
| 6 | (wellbeing\|well-being\|“quality of life”\|wellness) (“Micronesia" AROUND (4) “framework"\|”model”\|”theory”\|”knowledge”)\|(“Micronesian" AROUND (4) “framework"\|”model”\|”theory”\|”knowledge”)\|(“Palau" AROUND (4) “framework"\|”model”\|”theory”\|”knowledge”) | **Total:** ~17.5k  **New on pp. 1-8:** 75 |
| 7 | (wellbeing\|well-being\|“quality of life”\|wellness) (“Palauan" AROUND (4) “framework"\|”model”\|”theory”\|”knowledge”)\|(“Samoa" AROUND (4) “framework"\|”model”\|”theory”\|”knowledge”)\|(“Samoan" AROUND (4) “framework"\|”model”\|”theory”\|”knowledge”) | **Total:** ~1k |
| 8 | (wellbeing\|well-being\|“quality of life”\|wellness) (“Native" AROUND (4) “framework"\|”model”\|”theory”\|”knowledge”)\|(“Marshall Islands" AROUND (4) “framework"\|”model”\|”theory”\|”knowledge”)\|(“Marshallese" AROUND (4) “framework"\|”model”\|”theory”\|”knowledge”) | **Total:** ~240k |
| 9 | (wellbeing\|well-being\|“quality of life”\|wellness) (“Mariana islands" AROUND (4) “framework"\|”model”\|”theory”\|”knowledge”)\|(“Tahiti" AROUND (4) “framework"\|”model”\|”theory”\|”knowledge”)\|(“Tahitian" AROUND (4) “framework"\|”model”\|”theory”\|”knowledge”) | **NONE** |
| 10 | (wellbeing\|well-being\|“quality of life”\|wellness) (“Vanuatu" AROUND (4) “framework"\|”model”\|”theory”\|”knowledge”)\|(“ni-Vanuatu" AROUND (4) “framework"\|”model”\|”theory”\|”knowledge”)\|(“Melanesia" AROUND (4) “framework"\|”model”\|”theory”\|”knowledge”) | **Total:** ~18k |
| 11 | (wellbeing\|well-being\|“quality of life”\|wellness) (“Melanesian" AROUND (4) “framework"\|”model”\|”theory”\|”knowledge”)\|(“Polynesia" AROUND (4) “framework"\|”model”\|”theory”\|”knowledge”)\|(“Polynesian" AROUND (4) “framework"\|”model”\|”theory”\|”knowledge”) | **Total:** ~13k |
| 12 | (wellbeing\|well-being\|“quality of life”\|wellness) (“Oceania" AROUND (4) “framework"\|”model”\|”theory”\|”knowledge”)\|(“Pacific" AROUND (4) “framework"\|”model”\|”theory”\|”knowledge”)\|(“Pasifika" AROUND (4) “framework"\|”model”\|”theory”\|”knowledge”) | **Total:** ~25k |
