# Supplementary material for: A systematic scoping review for decolonial public and global health: Indigenous frameworks and models of wellbeing from Turtle Island and Moananuiākea
Source: Front Public Health. 2026 Jul 16;14:1809539. doi: 10.3389/fpubh.2026.1809539 (PMC13422500; doi:10.3389/fpubh.2026.1809539)
Supplement: Supplementary file 3 [file Table_3.DOCX]

**
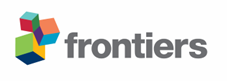
**

***Supplemental Material C. Decision Tree***

| Include if | Exclude if |
| --- | --- |
| - Describes a model, framework, or theory - Is about wellbeing, wellness, quality of life - Is for Indigenous adults, families, or communities - Target population is Indigenous to Turtle Island or Moananuiākea | - Not about wellbeing, wellness, quality of life - Doesn’t describe a model, framework, or theory - Not for adults, families, or communities (e.g., youth only) - Not for Indigenous people from Turtle Island or Moananuiākea - Specific to a health condition (e.g., COVID) - Focused on environmental wellbeing - Systematic review |
