# Supplementary material for: A systematic scoping review for decolonial public and global health: Indigenous frameworks and models of wellbeing from Turtle Island and Moananuiākea
Source: Front Public Health. 2026 Jul 16;14:1809539. doi: 10.3389/fpubh.2026.1809539 (PMC13422500; doi:10.3389/fpubh.2026.1809539)
Supplement: Supplementary file 4 [file Table_4.DOCX]

**
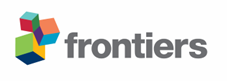
**

***Supplemental Material D. Data Extractions***

| Data Extracted | Definitions and Criteria |
| --- | --- |
| Model Name | The exact name of the model/framework/theory as the authors list it in the manuscript. |
| Discipline | Professional/academic discipline(s) which informed the development of the model/framework/theory. |
| Journal, Book, or Other | Type of publication. |
| Moananuiākea, Turtle Island | High-level geographic region - Moananuiākea (the Pacific) or Turtle Island (North America). |
| Place and Community | The geographic locale or Indigenous community the model/framework/theory was created by and/or intended to serve (e.g., Hawai`i, Native Hawaiians). |
| Type | The type of wellbeing “product” being introduced by the authors (e.g. framework, model).  Select all that apply:   - Model - Framework - Theory - Other (short text answer) |
| Study Participants | - Sample size - Gender - Role(s) of participants in relation to the larger community of study |
| Study Design | Select all that apply:   - Cross-Sectional - Survey - Observational - Document Review - Experimental - Qualitative (including storytelling) - Case study - Scoping review - Other (short text answer) |
| Aspects of Indigenous Wellbeing | Indication of various aspects of wellbeing located in the Model/Framework/Theory.  Select all that apply:   - Holism: approach or theory holding that a system or organism is a coherent, unified whole that cannot be fully explained in terms of individual parts or characteristics. The system or organism may have properties as a complete entity or phenomenon in addition to those of its parts. Thus, an analysis or understanding of the parts does not provide an understanding of the whole. - Relational(ality): views relationships as critical to wellbeing, including relationships to the spiritual realm, land, community, family, and oneself. - (Inter)connection: recognizes the interconnected nature of all. In particular, connection to place, sacred cycles of life, heritage. - Ecological: describes an ecology of being as the architecture of wellbeing connections. - Physical: describes aspects of physical wellbeing such as movement practices, or examples of healthy bodies - Psychological/Mental: describes thoughts (or thought patterns) or emotions associated with wellbeing - Collectivist: a social or cultural tradition, ideology, or personal outlook that emphasizes the unity of the group or community rather than each person’s individuality. Most Asian, African, and South American societies tend to put more value on collectivism than do Western societies, insofar as they stress cooperation, communalism, constructive interdependence, and conformity to cultural roles and mores. - Time as Cyclical, circular, or spiraled: as opposed to linear; e.g., the past is our future - Intergenerational: an emphasis on youth, adults, elders, perhaps gods and land all being connected for wellbeing - Family: not included as a level, but as a central part of the model; human, animal, land family (specify in subsequent qualitative question) - Community: could be neighborhood community, community of same tribe/ethnicity - Sovereignty: (Personal, Cultural, Political, Economic) - Land Stewardship - Land as Family - Balance: emphasizing harmony, resonance - Reciprocity: emphasizes the give and take of relationships, between people, land, animals, gods. - Culture: includes practices, speaking the language, engagement in activities or feeling of connection - Stories: names storytelling as a critical process within the wellbeing model, to ensure cultural sustainability, connection to elders. - Spirituality: as a dimension or underlying aspect; could include feeling connected to gods, ritual, values, - Material Quality of Life: this might include income or housing, for example - Supportive Determinants (e.g., cultural practice) - Detrimental Determinants (e.g., historical trauma) - Outcomes: description of what is the result of optimal wellbeing - Hedonic: relating to pleasure attainment, positive emotions, and pain avoidance (e.g., paahpilweeyankwi ‘we joke, are humorous’) - Eudaimonic: relating to meaning, purpose, and self-realization (e.g., neepwaahkaayankwi ‘we are wise, conscious, aware’) - Other (short text answer)   A second (long-form answer) field followed this variable, where data extractors could elaborate on the model/framework/theory’s features to capture information not included above.  Data extractors captured as accurately as possible the nomenclature used by the authors (e.g. "interconnected," "interdependent," and "relational" might be used interchangeably due to similar meanings, but data extractors captured the authors’ specific terminology). In addition, data extractors indicated whether certain features were explicitly named by the authors, or implicit in the data extractor’s interpretation of the model/framework/theory. |
| Imagery Used | Description of any visual imagery used by the authors to organize the model/framework/theory. |
| Organizing Categories | Description of the names and total # of organizing categories for the model/framework/theory. The authors’ specific nomenclature was used (e.g. “Dimension, Theme, Principle”).  Any sub-theme or sub-domains under the larger organizing categories were listed under “Organizing Category 2”. |
| Model Purpose | Authors’ stated purpose for creation of the model/framework/theory. |
| Future Directions | Any future directions suggested or indicated by the authors. |
| Definition of Wellbeing | Definition (1-3 sentences): Only if the authors provided an original definition of wellbeing OR explicitly cited their work as guided by other author(s)’ definition of wellbeing. |
| Values, Themes, or Guiding Principles | Collected if the authors indicated their work was *guided* by particular values, themes, or principles. Distinct, for example, from qualitative themes that may have *resulted* from the authors’ study.  As reported by team; summary of values and guiding principals listed below:   - Holistic approaches to wellbeing - Shared and collective responsibilities to promote wellbeing - Honoring cultural identities, belonging, and contexts related to intergenerational/historical trauma, strengths, and resilience - Honoring ancestral and generational knowledges, teachings, practices, and ways of being - Centering connection to land and the natural environment - Relational approaches to wellbeing by honoring sacred spaces, knowledge, and knowledge holders/keepers |
| Was a measure developed based on this work? | Yes, No, or Unsure |
| Additional Notes/Questions | Data extractors used this field to provide additional information (e.g., name of any measures developed), or for other analytic memos to discuss with the team during the quality assurance process. |
| Author Positionalities (Indigeneity) | Indicated whether at least one author identified as Indigenous (in the article or external publications), or if the model/framework/theory was at least created in close collaboration with an Indigenous community. |

*Initial features were selected by choosing a model or framework from Turtle Island and Moananuiākea to serve as examples; some features were added based on initial reviews of the literature.
